# Estrogen-Dependent Upregulation of *Adcyap1r1* Expression in Nucleus Accumbens Is Associated With Genetic Predisposition of Sex-Specific QTL for Alcohol Consumption on Rat Chromosome 4

**DOI:** 10.3389/fgene.2018.00513

**Published:** 2018-12-04

**Authors:** John Paul Spence, Jill L. Reiter, Bin Qiu, Hao Gu, Dawn K. Garcia, Lingling Zhang, Tamara Graves, Kent E. Williams, Paula J. Bice, Yi Zou, Zhao Lai, Weidong Yong, Tiebing Liang

**Affiliations:** ^1^Department of Medicine, Indiana University School of Medicine, Indianapolis, IN, United States; ^2^Comparative Medical Center, Institute of Laboratory Animal Science, Chinese Academy of Medical Sciences & Peking Union Medical College, Beijing, China; ^3^Greehey Children’s Cancer Research Institute, UT Health San Antonio, San Antonio, TX, United States; ^4^Department of Psychology, Southeast Missouri State University, Cape Girardeau, MO, United States

**Keywords:** alcohol use disorder, sex-difference, congenic rat model, nucleus accumbens, RNA-seq, *Adcyap1r1*

## Abstract

Humans show sex differences related to alcohol use disorders (AUD). Animal model research has the potential to provide important insight into how sex differences affect alcohol consumption, particularly because female animals frequently drink more than males. In previous work, inbred strains of the selectively bred alcohol-preferring (P) and non-preferring (NP) rat lines revealed a highly significant quantitative trait locus (QTL) on rat chromosome 4, with a logarithm of the odds score of 9.2 for alcohol consumption. Recently, interval-specific congenic strains (ISCS) were developed by backcrossing the congenic P.NP line to inbred P (iP) rats to further refine the chromosome 4 QTL region. Two ISCS sub-strains, ISCS-A and ISCS-B, were obtained with a narrowed QTL, where the smallest region of overlap consisted of 8.9 Mb in ISCS-B. Interestingly, we found that females from both ISCS lines consumed significantly less alcohol than female iP controls (*p* < 0.05), while no differences in alcohol consumption were observed between male ISCS and iP controls. RNA-sequencing was performed on the nucleus accumbens of alcohol-naïve female ISCS-B and iP rats, which revealed differentially expressed genes (DEG) with greater than 2-fold change and that were functionally relevant to behavior. These DEGs included down-regulation of *Oxt, Asb4, Gabre, Gabrq, Chat, Slc5a7, Slc18a8, Slc10a4*, and *Ngfr*, and up-regulation of *Ttr, Msln, Mpzl2, Wnt6, Slc17a7, Aldh1a2*, and *Gstm2*. Pathway analysis identified significant alterations in gene networks controlling nervous system development and function, as well as cell signaling, GABA and serotonin receptor signaling and G-protein coupled receptor signaling. In addition, β-estradiol was identified as the most significant upstream regulator. The expression levels of estrogen-responsive genes that mapped to the QTL interval and have been previously associated with alcohol consumption were measured using RT-qPCR. We found that expression of the *Adcyap1r1* gene, encoding the pituitary adenylate cyclase-activating polypeptide type 1 (PAC_1_) receptor, was upregulated in female ISCS-B compared to female iP controls, while no differences were exhibited in males. In addition, sequence variants in the *Adcyap1r1* promoter region showed a differential response to estrogen stimulation *in vitro*. These findings demonstrate that rat chromosome 4 QTL contains genetic variants that respond to estrogen and are associated with female alcohol consumption.

## Introduction

The development of alcohol use disorders has a strong genetic component. Genetic factors account for more than 50% of the variance in developing alcoholism (Heath et al., 1997; Ducci and Goldman, 2008), and several specific genetic variants are associated with an increased risk for alcoholism (Koss and Goldman, 2000; Dick et al., 2006; Gatti et al., 2010). In addition to genetics, gender-based differences in drug and alcohol use, abuse, and dependence are supported by epidemiological and clinical research (Prescott, 2002; Nolen-Hoeksema, 2004; Ceylan-Isik et al., 2010). Likewise, animal models also display sex-differences in voluntary consumption of drugs and alcohol (Vetter-O’Hagen et al., 2009; Desrivières et al., 2011). However, the mechanisms responsible for sex-specific drinking differences remain largely unknown (Becker and Koob, 2016).

Most sexually dimorphic traits arise through differential gene expression that has different effects on males and females (Dimas et al., 2012; Gershoni and Pietrokovski, 2017). In addition to phenotypic variation that results from the action of sex hormones, gene-by-sex (GxS) interactions involve phenotypic differences that depend on the functional genetic variants (Rawlik et al., 2016). Evidence for sex-specific quantitative traits exist that impact a wide range of complex traits (Karp et al., 2017). Sex-specific quantitative trait loci (QTLs) have also been reported for alcohol preference, ethanol sensitivity, and ethanol locomotor activation in mice and ethanol drinking in rats; however, specific genes mediating these effects have yet to be identified (Melo et al., 1996; Gill et al., 1998; Peirce et al., 1998; Radcliffe et al., 2000; Bice et al., 2006; Vendruscolo et al., 2006; Chesler et al., 2012; DuBose et al., 2013; Vanderlinden et al., 2015)

We and others have used the selectively bred alcohol-preferring (P) and non-preferring (NP) rat model (Li et al., 1991) to investigate the genetic factors involved in alcohol drinking behaviors (Bice et al., 1998; Carr et al., 1998; Liang et al., 2003, 2004; Liang and Carr, 2006). In this model, P rats exhibit several features that are consistent with alcoholism in humans (Cicero, 1979). For example, P rats (1) orally self-administer ethanol in pharmacologically relevant amounts; (2) consume EtOH for its pharmacological effects rather than caloric value or taste; (3) show positive reinforcement; (4) develop tolerance; and (5) exhibit withdrawal symptoms (McBride and Li, 1998; Murphy et al., 2002). QTL analysis of inbred alcohol-preferring/non-preferring (iP/iNP) rats revealed several loci associated with alcohol preference, including a region of chromosome 4 (Chr4) with a LOD score >9.2 (Carr et al., 1998; Bice et al., 2006).

Congenic rat strains were subsequently derived that had transferred the ∼ 130 Mb Chr4 QTL region of the donor strain to the host strain, whereby P.NP designates an iP host rat with the donor iNP Chr4 QTL, and NP.P designates an iNP host rat with the donor iP Chr4 QTL. Alcohol consumption in these reciprocal congenic strains were consistent with the donor strain, such that P.NP rats drank less than P rats and NP.P rats drank more than NP rats (Carr et al., 2006). These and other results confirmed the association of the Chr4 QTL with alcohol consumption, and indicated that multiple loci within this strong QTL may be contributing to the alcohol drinking phenotype (Carr et al., 2006; Spence et al., 2009; Liang et al., 2010). To further refine this Chr4 QTL region, overlapping interval-specific congenic strains (ISCS) were generated by backcrossing the P.NP congenic line with iP rats. This approach resulted in two ISCS lines (Spence et al., 2013). The ISCS-A sub-strain contained ∼ 79 Mb of the NP genomic region between microsatellite markers *D4Mgh16* and *D4Rat173*, while the ISCS-B sub-strain contained ∼ 9 Mb of the NP genomic region between a single nucleotide polymorphism (SNP) in *Snca* and the marker *D4Rat35* (Liang and Carr, 2006; Spence et al., 2013).

Similar to other animal models, female P and iP rats consume more alcohol than the corresponding males (Li et al., 1991; Carr et al., 1998). We hypothesized that a sex-specific QTL existed on rat Chr4 that contributed to the disparate alcohol drinking behavior between males and females. To test this hypothesis, we measured alcohol preference and consumption in male and female P.NP-ISCS rats since they contain a narrowed Chr4 QTL region. To investigate the genetic factors that might be involved in sex-specific drinking phenotypes, we also analyzed transcriptome differences between P.NP-ISCS-B and iP rats in the nucleus accumbens (NAc), a brain region known to play an important role in the reinforcing and rewarding effects of ethanol.

In this study, we report that a sex-specific QTL exists on rat Chr4 that contributes to alcohol consumption. Specifically, we found that replacement of an approximately 9 Mb region of the NP Chr4 locus into the iP genetic background decreased alcohol consumption in female, but not in male ISCS rats when compared to their respective controls. We report that RNA-seq analysis of NAc from female alcohol-naïve P.NP-ISCS and iP rats identified differentially expressed genes (DEGs) related to neuron function, cell signaling, and behavior. In addition, we found that β-estradiol was predicted to be the top upstream regulator of DEGs. Moreover, promoter SNPs in the Chr4 QTL gene *Acdyap1r1*, which encodes the PAC_1_ receptor for the neuropeptide pituitary adenylate cyclase-activating polypeptide (PACAP), responded to estrogen stimulation. These findings indicate that *Acdyap1r1*, a gene previously associated with alcohol abuse in women (Dragan et al., 2017), is a likely candidate gene contributing to female-specific drinking behavior.

## Materials and Methods

### Animals

Rats were bred and maintained at Indiana University School of Medicine. All animals were housed under 12-h light–dark conditions (7:00 am / 7:00 pm) with free access to laboratory rodent chow and water. The animals used for this study included inbred alcohol-preferring (iP) rats (Lumeng et al., 1977; Bice et al., 1998), congenic P.NP strains (Carr et al., 2006), and ISCS-A and -B (Spence et al., 2013). Heterozygous ISCSB-H F1 animals were generated by crossing ISCS-B with iP rats. The experimental protocol used in this study was reviewed and approved by the Indiana University Institutional Animal Care and Use Committee and was carried out in accordance with the NIH Guide for the Care and Use of Laboratory Animals. The animals were maintained in facilities fully accredited by the Association for the Assessment and Accreditation of Laboratory Animal Care (AAALAC).

### Alcohol Consumption and Preference in ISCS Sub-Strains

Adult alcohol-naïve male and female rats from the two P.NP-ISCS (A and B strains), ISCSB-H, and iP controls were tested for voluntary alcohol drinking using a 2-bottle free-choice protocol, consisting of 10% ethanol or water for 3 weeks, as described previously (Lumeng et al., 1977; Li et al., 1993). Littermates were included from both ISCS-A and ISCS-B strains when possible. Two experiments were conducted by comparing: 1) ISCS-A (23 males, 22 females) vs. iP controls (10 males, 15 females); and 2) ISCS-B (22 males, 23 females), ISCSB-H (20 males, 18 females), and iP control (23 males, 35 females). Both alcohol consumption and alcohol preference were calculated as was described previously (Li et al., 1991). Body weight, but not food intake, was recorded once a week. To exclude the possibility of a sex difference in taste reactivity, ISCS-B rats (8 females, 7 males) were first tested for saccharin (1.03%) and then for quinine (0.5 μM) intake. Consumption data was analyzed by two-way ANOVA followed by Newman-Keuls multiple comparisons test. Results are provided as means and standard error (SE).

### RNA Isolation From NAc

Whole brains were extracted from alcohol-naïve adult ISCS-B and iP control rats, and snap frozen in dry ice-bathed isopentane, before brain regions were dissected as previously published (Liang et al., 2004, 2010; Liang and Carr, 2006). The RNA-seq experiment used NAc from female ISCS-B and iP control rats (*N* = 3 for each group). Tissues were stored at -80°C until RNA isolation. RNA was isolated from the NAc using TRIzol, followed by RNeasy mini-column purification (Qiagen, Valencia, CA, United States). RNA purity was measured using a spectrophotometer (Nanodrop 1000) and the 260/280 absorbance ratios were between 1.8 and 2.0. RNA integrity was measured using an Agilent 2100 Bioanalyzer and all samples had RIN >7.

### RNA-seq Analysis

Sequencing libraries were constructed using the Illumina TruSeq RNA sample preparation protocol. The resulting libraries were sequenced on an Illumina HiSeq 2000 instrument using a standard single-end 50 bp sequencing protocol. The reads were aligned to the reference *Rattus norvegicus* genome (UCSC Rn 6.0) with TopHat 2 (Trapnell et al., 2010; Kim et al., 2013). No more than 2 mismatches were allowed in the alignment. HTseq was used to count gene expression reads, and DEseq was used to find DEGs after performing median normalization (Anders and Huber, 2010; Anders et al., 2015). DEGs were identified with adjusted *p* < 0.05 for multiple tests by the Benjamini-Hochberg method for controlling false discovery rate (FDR; Benjamini and Hochberg, 1995) and using a cutoff of sequence read rpmk >1. The RNA-seq data is available at GEO^1^ with access ID: GSE112399.

### IPA and Reactome Pathway Analysis

Two pathway analyses were used in data exploration. Ingenuity Pathway Analysis (IPA), which builds on manually curated content of the Ingenuity Knowledge database, was applied for predicting significant biological mechanisms and pathways (Qiagen^2^). The complete data set containing gene identifiers and corresponding expression values was uploaded into the application. Each identifier was mapped to its corresponding object in Ingenuity’s Knowledge Base. An FDR-adjusted *p*-value cutoff of 0.05 was set to identify molecules whose expression was significantly differentially regulated. These molecules, called Network Eligible molecules, were overlaid onto a global molecular network developed from information contained in Ingenuity’s Knowledge Base. Networks of Network Eligible Molecules were then algorithmically generated based on their connectivity. The Functional Analysis identified the biological functions and/or diseases that were most significant to the data set. Molecules from the dataset that met the FDR-adjusted *p*-values cutoff of <0.05 and were associated with biological functions and/or diseases in Ingenuity’s Knowledge Base were considered for the analysis. Right-tailed Fisher’s exact test was used to calculate a *p*-value determining the probability that each biological function and/or disease assigned to that data set is due to chance alone. Canonical pathways analysis identified the pathways from the Ingenuity Pathways Analysis library of canonical pathways that were most significant to the data set. Molecules from the data set that met the FDR-adjusted *p*-values cutoff of *p* <0.05 and were associated with a canonical pathway in Ingenuity’s Knowledge Base were considered for the analysis. The significance of the association between the data set and the canonical pathway was measured in two ways: (1) A ratio of the number of molecules from the data set that map to the pathway divided by the total number of molecules that map to the canonical pathway is displayed. (2) Fisher’s exact test was used to calculate a *p*-value determining the probability that the association between the genes in the dataset and the canonical pathway is explained by chance alone.

In addition, the *Reactome* Knowledgebase version 62^3^ was applied to analyze the molecular interaction details of signal transduction, transport, and other cellular processes (Fabregat et al., 2016). Settings for *Reactome* analysis were DEGs with *p*-values <0.05 and FC > 2; a total of 212 genes were used for analysis. Pathway hierarchical organization provides *Reactome* pathways overview and highlights parent-child relationships of overrepresented connections in the pathway.

### Validation of Candidate Gene Expression

Reverse transcription quantitative PCR (RT-qPCR) was performed using RNA from a separate set of experimental male and female ISCS-B and iP control (*N* = 6–8) animals than were utilized for RNA-seq. Each PCR assay was conducted using 6–8 biological replicates, and each cDNA sample was amplified in triplicate in qPCR for the same RT reaction. In brief, 1 μg RNA was reverse transcribed using Superscript III reverse-transcription reagent for first-strand cDNA synthesis (Invitrogen) using random primers. Each 50 μl PCR reaction contained cDNA corresponding to 35 ng of total RNA, SYBR Green Real-Time PCR Master mix (Life Sciences), and primers (5 μM). PCR was performed using the ABI PRISM 7300 Sequence Detection System (ThermoFisher), relative mRNA expression levels were normalized to *Gapdh*, and the standard curve method was used for data analysis. *Vector NTI* was used for primer design, and annealing temperatures ranged from 60 to 63°C. The primers used are listed in Supplementary Table S5. *T*-test was used to analyze the data and statistical significance was set at *p* < 0.05.

### DNA Sequence Analysis

Genomic DNA was isolated from iP and iNP rats using Gentra Puregene Tissue kit (QIAGEN). PCR primers were designed to amplify the *Adcyap1r1* promoter region up to 2 kb upstream of the transcription start site; primer sequences are listed in Supplementary Table S5. PCR products were purified using the GenElute PCR Cleanup Kit (Sigma, St. Louis, MO) and ligated upstream of the luciferase gene in the pGL3-basic vector (Invitrogen). Plasmid DNA was isolated using the QIAprep Spin Miniprep kit, and the cloned *Adcyap1r1* promoter was sequenced using Indiana University sequencing core service. Genomic DNA sequences were aligned to the Rn.6 reference sequence.

### Transient Transfection and Dual-Luciferase Activity Assays

The P and NP reporter plasmids utilized in this study were constructed as previously described (Liang et al., 2003). Human neuroblastoma SK-N-SH cells (ATCC HTB-11) were cultured in Minimal Essential Medium (Invitrogen) containing 7.5% NaHCO_3_, 2 mM Glut-max, 0.1 mM non-essential amino acids, 1 mM pyruvate, and 10% FBS (Invitrogen, Carlsbad, CA, United States) at 37°C in a humidified 5% CO_2_ incubator. Twenty-four hours before transfection, 5.0 × 10^4^ cells were plated into individual wells of a 24-well plate. Using Tf_x_-50 reagent (Promega, Madison, WI, United States), each well was co-transfected with 0.5 μg of the pGL-3 luciferase plasmid plus 4 ng of the CMV Renilla luciferase vector (pRL-CMV, Invitrogen), which was utilized as an internal control for transfection efficiency. The human ER-α expression vector was a kind gift from Dr. Edwin R. Sánchez. The cells were subsequently incubated for 24 h at 37°C and washed with PBS before cell extracts were prepared in passive lysis buffer and assayed for firefly and Renilla luciferase activities using the Dual-Luciferase Reporter Assay System (Promega) in a TD-20/20 Luminometer (Turner BioSystems). The reporter assays were repeated five times in triplicate using plasmids that were independently purified at least twice.

## Results

### Recessive Sex-Specific QTL Maps to Rat Chromosome 4

**FIGURE 1:**
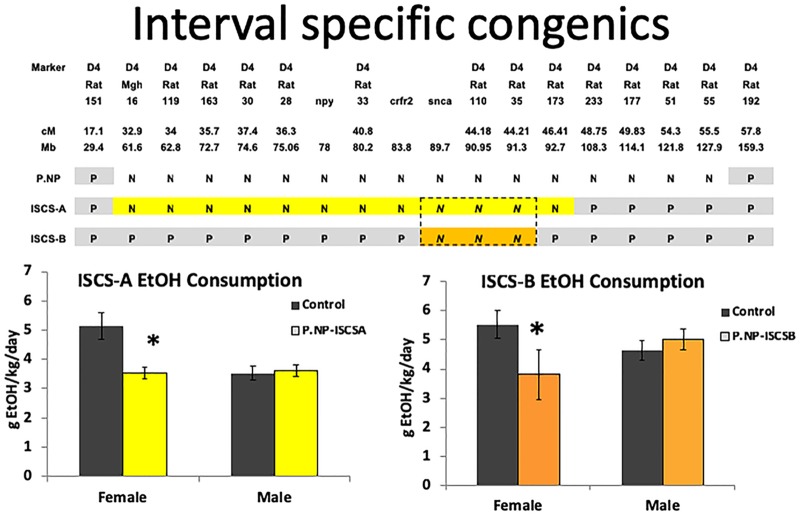
P.NP is the congenic strain with the Chr4 QTL region in P rats replaced by the NP Chr4. Backcrossing P.NP with iP rat resulted in interval-specific congenic strains (ISCS). ISCS-A and ISCS-B shared a narrowed Chr4 QTL between 83.8 and 92.7 Mb (region in dash-lined box). Relative to iP control rats, only females in either ISCS consumed less alcohol; alcohol consumption (gEtOH/Kg/day) was calculated and is shown as mean ± SEM. ^∗^indicates *p* < 0.05.

We examined whether the narrowed Chr4 QTL in the ISCS-A and ISCS-B sub-strains affected the drinking phenotype by testing alcohol consumption. In most alcohol drinking animal models, females have higher alcohol consumption than male rats, and this same phenotype was observed in control iP rats (Figure 1). However, we found that alcohol consumption was similar between males and females of both ISCS rat lines (∼3.5 to 4.5 g EtOH/kg BW/day). When ISCS alcohol consumption was compared to the iP background strain, both female ISCS-A and ISCS-B rats consumed 20–30% less alcohol (*p* < 0.05), while no differences were observed in the male rats (Figure 1). Our findings suggest a sex-specific QTL that maps to the overlapping Chr4 region in these two sub-strains between approximately 83.8 and 92.7 Mb (position reference to RGSC-v3.4).

To determine whether female alcohol consumption was a dominant or recessive trait, we tested alcohol consumption in heterozygous offspring of the ISCS-B line since this sub-strain contained the smallest overlapping genomic region. We found female homozygous ISCS-B animals consumed less alcohol than female heterozygous ISCSB-H (*p* < 0.05); however, we found no difference between female homozygous iP control and ISCSB-H. The similarity in alcohol consumption between iP and ISCSB-H indicates that the reduction in female drinking is a recessive trait. Additionally, no difference was detected in alcohol consumption between males of any genotypes (Supplementary Figure S1). No sex differences were observed with either saccharin (adjusted *p-*value = 0.6) or quinine intake (adj *p* = 0.56), which indicates that differences in alcohol consumption were not associated with taste preferences (Supplementary Figure S2). Furthermore, alcohol consumption was not associated with body weight since we previously showed that there were no differences in body weight between ISCS-B and iP animals (Spence et al., 2013).

### Identification of DEGs in the NAc Between ISCS-B and iP Rats

Since the only genetic differences between ISCS-B and iP rats existed within a minimum 1.79 Mb and maximum 8.9 Mb region of Chr4, we reasoned that genes in this narrowed Chr4 QTL might impact global gene expression in female rats related to alcohol consumption. To identify transcriptome changes, we performed RNA-seq on the NAc of alcohol-naïve female ISCS-B and iP (control) rats. High quality RNA-seq data were generated as documented by the correlation of biological replicates higher than 0.96. The average read count of each sample was more than 42 million with an average of more than 99.9% mapped reads (Supplementary Table S1).

We found 759 DEGs with the FDR set at *p* < 0.05; 212 of these DEGs showed FC > 2. The top 30 up- and down-regulated genes with adj *p* < 0.05 in ISCS-B females are listed in Table 1. Among the down-regulated genes, some are important for neuron functions: *Oxt*, and *Gabre* (Donhoffner et al., 2016). Four genes were involved in the cholinergic system: *Chat, Slc5a7, Slc18a3*, and *Lhx8*. Other significant down-regulated genes are known to influence neurotransmission, neuron growth, and addiction, including *Ngfr, Ntrk1*, and *Ntsr1* (Gehle and Erwin, 1998; Pandey et al., 2017); and the estrogen receptor gene *Esr1* is an important regulator of sex-differences. Among the up-regulated genes, *Wnt6 and Cdh1* are associated with neuron development; other up-regulated DEGs are related to detoxification of drug and alcohol metabolism, such as *Ptgds* and *Gstm2*. Notably, many of the up-regulated DEGs are related to the development and function of the nervous system. The complete list of DEGs with FC > 2 and FDR adj *p* < 0.05 can be found in Supplementary Table S3.

**Table 1:** Top 30 up- and down-regulated genes.

Gene symbol	Full name	log2 fold change	adj-*p*-value
**Top molecules down-regulated**			
*Oxt*	Oxytocin/neurophysin 1 prepropeptide	-8.582	0.019
*Tmem212*	Transmembrane protein 212	-4.604	0.003
*Gabre*	Gamma-aminobutyric acid (GABA) A receptor; epsilon	-3.494	0.017
*Ngfr*	Nerve growth factor receptor	-3.321	0.000
*Spata18*	Spermatogenesis associated 18	-3.043	0.009
*Mir384*	microRNA 384	-2.644	0.007
*Lhx8*	LIM homeobox 8	-2.427	0.000
*Wdr63*	WD repeat domain 63	-2.308	0.045
*Slc18a3*	Solute carrier family 18 member A3	-2.164	0.000
*Slc5a7*	Solute carrier family 5 (sodium/choline cotransporter); member 7	-2.087	0.000
*Esr1*	Estrogen receptor 1	-2.064	0.013
*Chat*	Choline O-acetyltransferase	-2.004	0.000
*Slc10a4*	Solute carrier family 10; member 4	-1.997	0.000
*Klhl1*	Kelch-like family member 1	-1.989	0.003
*St8sia6*	ST8 alpha-N-acetyl-neuraminidase alpha-2;8-sialyltransferase 6	-1.964	0.008
*Ntrk1*	Neurotrophic tyrosine kinase; receptor; type 1	-1.962	0.000
*Gpr165*	G protein-coupled receptor 165	-1.900	0.001
*Zim1*	Zinc finger; imprinted 1	-1.853	0.000
*Stk32b*	Serine/threonine kinase 32B	-1.835	0.000
*Ecel1*	Endothelin converting enzyme-like 1	-1.733	0.000
*Dynlrb2*	Dynein light chain roadblock-type 2	-1.722	0.031
*Ntsr1*	Neurotensin receptor 1	-1.713	0.000
*Gbx1*	Gastrulation brain homeobox 1	-1.708	0.000
*Ppp1r32*	Protein phosphatase 1; regulatory subunit 32	-1.682	0.000
*Pth2r*	Parathyroid hormone 2 receptor	-1.661	0.000
*Shh*	Sonic hedgehog	-1.625	0.000
*Slc27a2*	Solute carrier family 27 (fatty acid transporter); member 2	-1.624	0.002
*Lrrc34*	Leucine rich repeat containing 34	-1.624	0.001
*Nkx2-1*	NK2 homeobox 1	-1.617	0.001
*Gpx3*	Glutathione peroxidase 3	-1.608	0.014
**Top molecules up-regulated**			
*Rn5-8s*	5.8S ribosomal RNA	2.898	0.019
*LOC310926*	hypothetical protein LOC310926	2.247	0.001
*Mpzl2*	myelin protein zero-like 2	1.945	0.000
*LOC100134871*	beta globin minor gene	1.923	0.037
*LOC689064*	beta-globin	1.849	0.014
*Shisa3*	shisa family member 3	1.797	0.001
*Ccl9*	chemokine (C-C motif) ligand 9	1.783	0.025
*Aqp1*	aquaporin 1	1.744	0.000
*Cxcl10*	chemokine (C-X-C motif) ligand 10	1.698	0.034
*Wnt6*	wingless-type MMTV integration site family; member 6	1.663	0.000
*Gstm2*	glutathione S-transferase mu 2	1.637	0.011
*Osr1*	odd-skipped related transciption factor 1	1.550	0.000
*RT1-Da*	RT1 class II; locus Da	1.545	0.000
*Cd74*	Cd74 molecule; major histocompatibility complex; class II invariant chain	1.544	0.000
*Folr2*	folate receptor 2 (fetal)	1.541	0.024
*Cdh1*	cadherin 1	1.540	0.023
*Kcnj13*	potassium channel; inwardly rectifying subfamily J; member 13	1.503	0.000
*Gjb2*	gap junction protein; beta 2	1.494	0.000
*Tfap2b*	transcription factor AP-2 beta	1.489	0.004
*Ptgds*	prostaglandin D2 synthase (brain)	1.463	0.000
*RGD1305645*	similar to RIKEN cDNA 1500015O10	1.457	0.002
*Clec10a*	C-type lectin domain family 10; member A	1.451	0.000
*Cyp1b1*	cytochrome P450; family 1; subfamily b; polypeptide 1	1.449	0.010
*PCOLCE2*	procollagen C-endopeptidase enhancer 2	1.447	0.000
*Bhlhe22*	basic helix-loop-helix family; member e22	1.440	0.000
*Slc22a6*	solute carrier family 22 (organic anion transporter); member 6	1.438	0.000
*Rln1*	relaxin 1	1.423	0.006
*Serpinf1*	serpin peptidase inhibitor; clade F (alpha-2 antiplasmin; pigment epithelium derived factor); member 1	1.402	0.001
*Slfn3*	schlafen 3	1.394	0.000
*Chrnb3*	cholinergic receptor; nicotinic; beta 3 (neuronal)	1.363	0.002


*DEG between ISCSB and iP control female rats in the nucleus accumbens with highest fold change and with FDR adjusted *p* < 0.05 are listed.*

**FIGURE 2:**
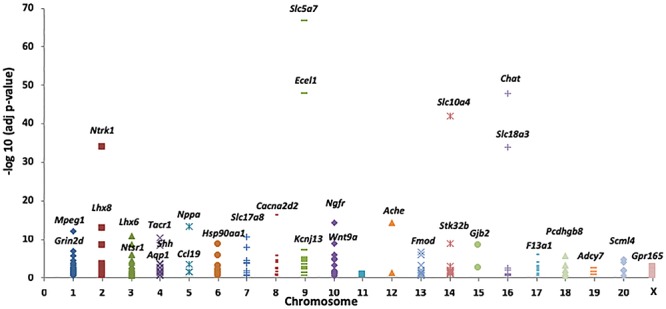
A total of 73 differentially expressed genes (DEG) between female iP and ISCS-B rats possess a fold change >2 with *p* < 0.05. The significance of these genes was plotted by corresponding chromosomes. Genes with the lowest *p*-values for each chromosome are labeled.

To determine the genomic distribution of the DEG identified in the present study, we plotted the negative log-adjusted *p*-values for each DEG by chromosome (Figure 2). Of the 212 DEGs with FC > 2 (Supplementary Table S3), we found that 13 genes mapped to Chr4, including *Tacr1, Chrm2, Neurod6, Slc13a4*, and *Aqp1*; notably, these genes were differentially expressed and associated with drinking differences. Among the Chr4 DEG, *Tacr1*, which encodes the receptor for tachykinin substance P (also known as neurokinin 1), has been found to be associated with alcohol consumption in both humans and animals (Thorsell et al., 2005; George et al., 2008; Schank et al., 2013).

### Networks Associated With DEGs in NAc and Pathway Analysis of *Trans*-Acting Factors That Affect Sex-Specific Gene Expression

**FIGURE 3:**
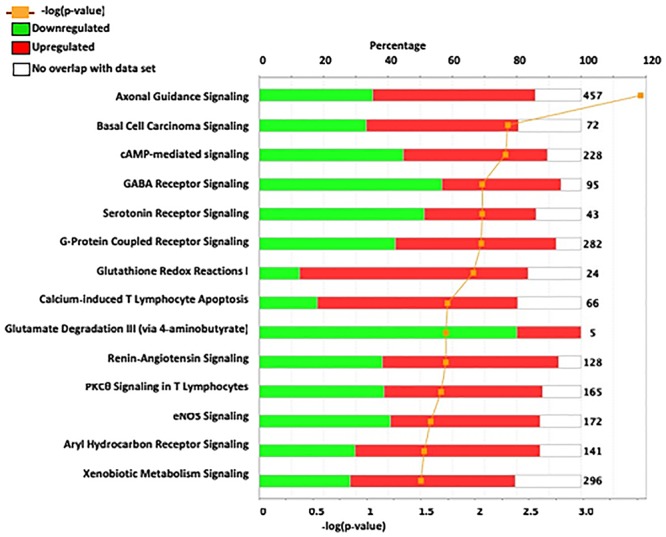
In depth analysis of DEG between ISCS-B and iP using IPA. The most significant IPA canonical pathways are listed on the left. The stacked bar chart reveals the percentage of up-regulated (red) and down-regulated (green) genes within each canonical pathway. The numerical value at the right of each bar represents the total number of genes in the canonical pathways. The secondary x-axis (bottom) represents the -log of *p*-value.

We utilized IPA to predict relevant molecular networks, biological functions, and canonical pathways altered between ISCS-B and iP female rats. The input gene list included 790 DEGs with an FDR-adjusted *p*-value cutoff of 0.05 (Figure 3 and Supplementary Figure S3). The most significant networks identified were relevant to axonal guidance signaling, cAMP-mediated signaling, and GABA receptor signaling, which are also important for drinking behavior (Figure 3). Genes within these pathways that have been associated with alcohol consumption in previous research studies include *Adcy7, Aldh1a2, Chrm2, Grin2d, Nfkb, Gsat4, Tacr1*, and *Oprm1*. Interestingly, the majority of genes in GABA receptor signaling and glutamate degradation III pathways showed reduced expression, while more genes with increased expression were found in pathways involved in axonal guidance, G-protein coupled receptor signaling, and glutathione-redox reactions (Figure 3).

To understand what factors might be driving these DEGs, we used IPA upstream analysis to predict the top transcriptional regulators. The top three upstream regulators were β-estradiol, α-synuclein, and β-catenin. Other additional relevant upstream regulators are included in Table 2. Among these predicted upstream regulators, our findings suggest that the hormones β-estradiol, progesterone, as well as dihydrotestosterone, likely contribute to differences in sex-specific gene expression.

**Table 2:** Upstream regulators.

Upstream regulator	Full name	Activation *z*-score	*p*-value of overlap
Beta-estradiol	Beta-estradiol	-0.563	1.78E-23
SNCA	α-synuclein	3.667	2.27E-12
CTNNB1	Catenin beta 1	0.807	1.47E-11
Progesterone	Progesterone	1.34	2.95E-11
HTT	Huntingtin	0.808	7.94E-11
CREB1	cAMP responsive element binding protein 1	0.952	1.03E-10
JAK1/2	Janus kinase 1	4.123	1.74E-10
BDNF	Brain derived neurotrophic factor	-1.374	8.24E-10
JUN	Jun proto-oncogene, AP-1 transcription factor subunit	1.345	1.57E-09
IFNG	Interferon gamma	2.602	8.33E-09
Dihydrotestosterone	Dihydrotestosterone	0.89	2.37E-08
ESR1	Estrogen receptor 1	-0.193	3.36E-08


### Reactome Pathways and Overrepresented Connections in the Pathway

**FIGURE 4:**
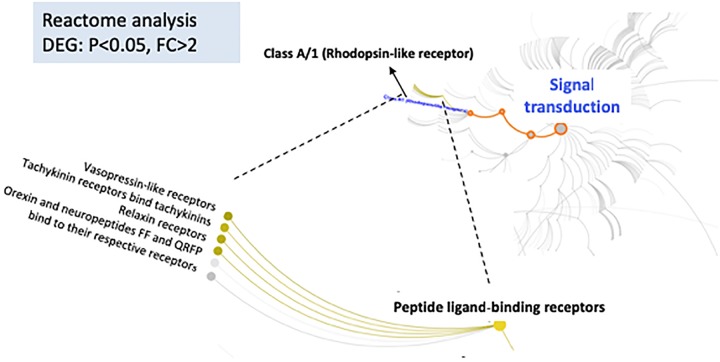
Reactome database was used to analyze DEG relationships organized into biological pathways and processes (https://reactome.org/dev/). It reveals significant alterations in signal transduction pathways between female iP and ISCS-B rats. Color-highlighted edges indicate over-represented interactions.

In addition to IPA analysis, DEGs with *p*-values <0.05, FC > 2 were used as input for *Reactome* data analysis, which is focused on molecular interactions within cells. The results of this analysis showed that the signal transduction pathways involving peptide ligand-binding receptors of class A/1 had the lowest *p*-values, indicating the significance of these interactions (Figure 4). We also found interesting regulator effect networks in our dataset. For example, Ngf and Raf1 play roles in dopamine and coordination, and ASCL1, estrogen receptor, and NGF are regulators of cell movement in neurons. More networks can be found in the (Supplementary Table S4).

### Significant DEGs in NAc Between Males and Females Comparing ISCS-B and iP Rats

**FIGURE 5:**
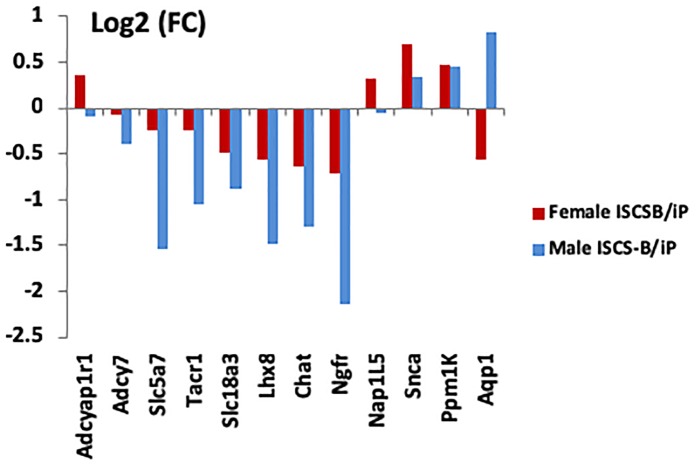
RT-qPCR comparisons of gene expression between male and female iP and ISCS-B rats. *Chat, Slc18a3, Lhx8*, and *Ngfr* were confirmed to be differentially expressed in both male and female ISCS-B compared to iP rats. *Adcyap1r1* and *Nap1L5* were upregulated in female ISCS-B compared to iP animals, but no difference was found in males.

**FIGURE 6:**
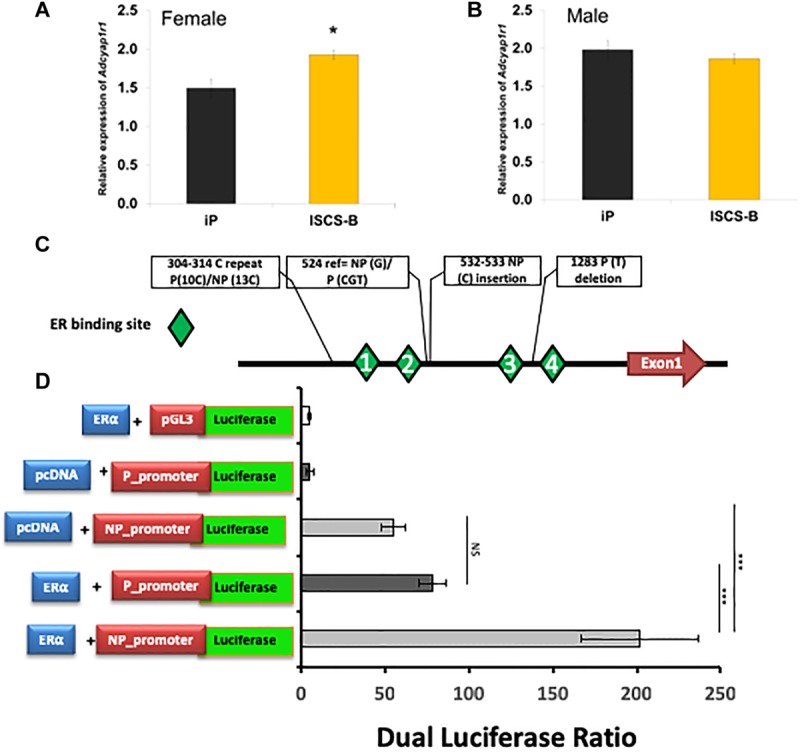
**(A)** *Adcyap1r1* expression is increased in female ISCS-B relative to iP rats. **(B)** Male ISCS-B rats demonstrate no significant difference from iP in *Adcyap1r1* expression. **(C)** A schematic of the *Adcyap1r1* promoter sequence demonstrating ER binding sites and polymorphisms between iP and iNP. **(D)** ERα transactivation of both variants of the *Adcyap1r1* promoter enhanced luciferase activity, but the iNP promoter exhibited significantly more luciferase activity than iP.

To determine whether the DEG identified in female NAc were sex-specific or also were differentially expressed in males, mRNA expression levels of selected DEGs were measured using RT-qPCR in both male and female ISCS-B and iP NAc tissue. The genes selected for measurement either showed a high fold change between the ISCS-B and iP RNA-seq data (e.g., *Lhx8, Ngfr, Slc5a7*), or were located in the Chr4 QTL and had been shown to be associated with alcohol consumption (e.g., *Adcyap1r1, Adcy7, Snca*, and *Tacr1*) or were reported previously to be differentially expressed between P and NP rats (e.g., *Ppm1K* and *Aqp1*). RT-qPCR was performed using RNA isolated from a different set of ISCS-B and iP rats. Figure 5 displays the fold-change (ISCS-B/iP ratio) and Supplementary Table S2 includes relative gene expression levels and *t*-test *p*-values between ISCS-B and iP animals for both males and females. Among these genes, *Chat, Lhx8, Ngfr, Slc18a3*, and *Tacr1* were found to be differentially expressed in both male and female ISCS-B compared to iP rats; however, differential expression of *Adcy7* and *Slc5a7* was observed between ISCS-B and iP males (*p* = 0.009 and 0.002, respectively), but no differences were found in females. Interestingly, *Nap1l5*, which is a maternally imprinted gene (Smith et al., 2003), was up-regulated in ISCS-B compared to iP females, but not males, which suggests that altered expression might be related to sex-specific differences in epigenetic regulation. Of particular relevance, *Adcyap1r1*, which has been associated with drinking in women (Dragan et al., 2017), was also up-regulated in ISCS-B compared to iP females, but was not significantly different in males (Figures 6A,B).

### Rat *Adcyap1r1* Promoter Contains Predicted Estrogen-Response Elements

In addition to RNA-seq, we also performed GenBank and literature searches for genes that mapped to the Chr4 interval region and that had evidence of an association with alcohol consumption or response to estrogen. This approach identified several estrogen-responsive genes, including *Adcyap1r1*. Importantly, the *Adcyap1r1* genotype was associated with alcohol abuse in women (Dragan et al., 2017), and it also has been shown to respond to alcohol treatment (Koh et al., 2006). In addition, the human *ADCYAP1R1* gene contains a SNP in an estrogen response element (ERE) that was associated with PTSD only in females (Ressler et al., 2011). Therefore, we selected this gene for further mechanistic investigation to determine whether an ERE also existed in the rat *Adcyap1r1* promoter, and if any genetic variants existed between iP and iNP strains that might affect estrogen receptor α (ERα) binding. Estrogen receptor binding site prediction was performed using Genomtix^4^, which revealed four potential EREs within 2 kb upstream of the *Adcyap1r1* transcription start site (Figure 6C), which was designated as + 1. We cloned and sequenced a 2 kb region of the *Adcyap1r1* promoter from genomic DNA of both iP and ISCS-B, which contained the *Adcyap1r1* iNP Chr4 sequence. In addition to confirming the presence of ERE consensus sites, we identified five sequence variants, including two that mapped near predicted EREs at nt 485–494 (#1) and nt 1303–1310 (#4) (Table 3 and Figure 6).

**Table 3:** Promoter variance in *Adcyap1r1* gene.

	304–314 repeat C	524	532–533	1283	1885–1886
ReqSeq	C (11)	G	-	T	-
iP	C (10)	CGT	-	-	A
iNP	C (13)	G	C insertion	T	G


*The nt location reference to 2 kb (designate as + 1) upstream of TSS.*

### Luciferase Expression of iNP Promoter of *Adcyap1r1* Was Significantly Upregulated by ERα

To evaluate the potential of these genomic variants to act as *cis*-elements in ERα transactivation of *Adcyap1r1* gene expression, we performed *in vitro* dual luciferase promoter assays. The iP and iNP promoter regions of *Adcyap1r1* were cloned into the pGL3 vector separately, and tested with and without dual ERα expression from the pcDNA vector. When empty pcDNA vector was transfected, the NP promoter showed higher luciferase activity compared to the P promoter. This effect could result from an endogenous estrogen effect or from the action of other transcription factors. As shown in Figure 6, luciferase expression was significantly upregulated by ERα, when the *Adcyap1r1* promoter was transcribed from the iNP compared to the iP genome. This result indicated that the iNP variant was more sensitive to ERα binding than the iP variant and suggested that these variants could contribute to the observed sex-differences in alcohol consumption between the iP and iNP strains.

## Discussion

Our results revealed a sex-specific QTL for alcohol consumption on rat Chr4 and point to a potential role for the gene *Acdyap1r1* in female-specific alcohol drinking behavior in the P rat model of AUD. Specifically, we identified DEG’s in the NAc that were associated with the transferred QTL region in ISCS-B rats. These DEG’s were predicted to affect gene networks controlling nervous system development and function, as well as drug metabolism and behavior. Notably, the top predicted upstream regulator of DEG’s in female NAc was β-estradiol. Importantly, we found that sequence variants in the *Acdyap1r1* promoter showed differential activation by the estrogen receptor.

Females drink more alcohol than males in most, if not all, selectively bred lines of rodent models for alcohol consumption, including the iP rats used in this study (Li and Lumeng, 1984; Lancaster and Spiegel, 1992; McBride and Li, 1998; Chester et al., 2006). In the present study, we found that when a relatively small region of the iNP genome was substituted in the iP Chr4 QTL region, homozygous female ISCS rats consumed less alcohol (g EtOH/kg BW/day) and they exhibited similar alcohol consumption compared to iP control animals (Figure 1); this effect was not detected in their male counterparts. The reduced alcohol consumption in ISCS females was not due to body weight since we previously demonstrated that ISCS-B animals do not differ in body weight from the iP line (Spence et al., 2013). Additionally, because heterozygous female ISCSB-H animals consumed similar amounts of alcohol as iP controls, the sex-specific QTL we identified is a recessive trait.

The sex-specific drinking phenotype identified in the current research suggests that genetic variation from the NP genome within the ISCS-B congenic region likely contributes to decreased alcohol consumption in females in the P and NP model. We previously created the ISCS lines that decreased the background heterogeneity found in the P.NP congenic strain and simultaneously narrowed the QTL region (Spence et al., 2009; Spence et al., 2013; Baud and Flint, 2017). The sole difference within the entire genomes of ISCS-B and iP is a 1.7–8.9 Mb genomic segment, or less than approximately 0.3% of the 2719 Mb rat genome (Jensen-Seaman et al., 2004). Clearly, this ISCS-B genomic region contains variants from the NP donor, which modify gene expression via *cis*-elements or interact with *trans*-acting factors (e.g., estrogen receptor), thereby altering gene networks that significantly affect female alcohol consumption (Figure 6). We hypothesized that many interactions exist between genes within this 1.7–8.9 Mb genomic region and genes outside this region, and replacement of this region would result in *trans*-regulation via *cis-acting* elements.

**FIGURE 7:**
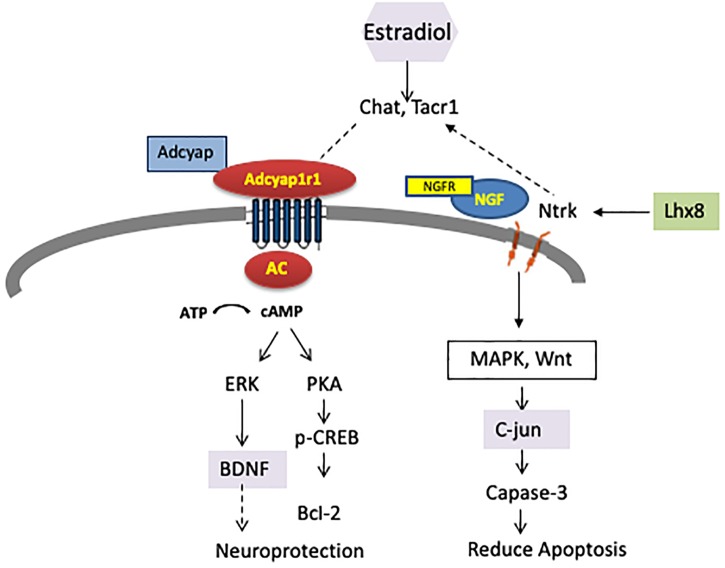
DEGs were enriched in two pathways which affect neuron function and are also regulated by estrogen receptor (ER). Adenylate cyclase (AC) and downstream signaling affecting neuroprotection was upregulated, while NtrK, Ngfr, and Lhx8 signaling affecting apoptosis were downregulated.

*Adcy7* has been associated with female-specific ethanol consumption in mice (Pronko et al., 2010; Cruz et al., 2011; Desrivières et al., 2011). IPA showed that *ADCY7, ADCY*, and *Creb* are involved in drug metabolism and behavior. Our finding indicated that the adenylate cyclase (AC) signaling pathway was enriched with genes associated with female alcohol consumption or response to estrogen regulation (Figure 7). We emphasize that upregulation of *Adcyap1r1* may affect AC, and downstream genes such as *PAK, ERK* and *BDNF*, which could also play important roles in neuron protection.

Hormones affect gene expression at both *cis-* (e.g., in the narrowed Chr4 region) and *trans*- (elsewhere) locations. Through data mining and literature searching, we identified multiple genes in the Chr4 QTL (e.g., *Aqp1, Abcg2, Adcyap1r1, Npy*, and *Snca*) that are regulated by estrogen directly or indirectly. Bioinformatics analysis also predicted upstream regulators of the DEGs which indicated that sex hormones are likely to play an important role (Table 3). Hormones, such as β-estradiol, target multiple DEGs, including *Tacr1* on Chr4, and *Chat, Nts, Nupr1, Ogn, Ttr*, and *Oprm1* on other chromosomes. Importantly, all these genes have relatively high fold change between ISCS-B and iP rats. In addition, β-estradiol is also predicted to target *Aldh1a2* and affect alcohol metabolism. *Esr1* encoding the estrogen receptor α is significantly decreased in ISCS-B NAc. Interestingly, β-estradiol is a regulator of the *Chat* and *Tacr1* genes.

**FIGURE 8:**
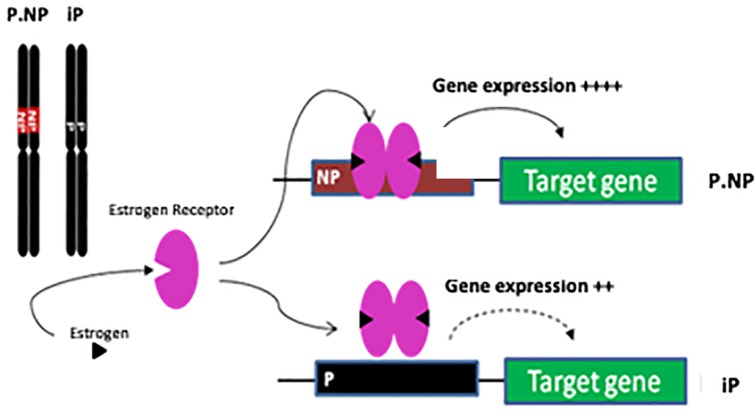
Schematic of genetic and estrogen receptor interaction affecting gene expression. The only difference between congenic P.NP and iP is on Chr4 region. Compared with P genomic sequence, the genetic difference in NP is proposed to lead to stronger binding of estrogen receptor in promoters of targeted genes resulting in increased gene expression.

We hypothesized that the sex-dependent differences in gene expression were related to promoter variants between the iP and ISCS-B lines, altering the binding affinity for *trans-acting* factors (e.g., estrogen receptor) involved in gene transcription (Figure 8). We show that genetic variants in the *Adcyap1r1* promoter respond to estrogen stimulation, thereby providing one possible mechanism for sex-specific differences in alcohol consumption. In humans, *ADCYAP1R1* has been associated with female alcohol consumption (Dragan et al., 2017). Pituitary adenylyl cyclase-activating polypeptide (ADCYAP)-ADCYAP1R1 pathway is regulated by estrogen and is involved in abnormal fear responses underlying PTSD (Ramikie and Ressler, 2016). Our finding that SNPs in the promoter region of the NP *Adcyap1r1* allele are more sensitive to estrogen stimulation *in vitro* suggests that alcohol consumption is mediated, in part, by estrogen regulation of ADCYAP1R1 in the P and NP model, providing a potential explanation for why alcohol consumption was decreased in female ISCS lines. Moreover, ADCYAP was found to be co-localized with ChAT in nerve fibers (Drescher et al., 2006) and it is possible that *Adcyap1r1* could also interact with ChAT, thereby providing a potential mechanism for how increased *ADCYAP1R1* expression might promote neuroprotection (Figure 8). In addition, pathway analysis showed an increase of glutathione-redox reactions I, conveying a similar neuroprotection function.

In this research, we observed that genes related to cholinergic function were reduced more than 4-fold in ISCS-B relative to iP, including *Chat, Slc18a3, Slc5a7*, and LIM homeobox 8. Consistent with our findings, previous studies found higher expression of *Chat, Slc18a3*, and *Slc5a7* in the NAc shell of adult P rats compared to NP rats (McBride et al., 2013). Further, cocaine treatment and withdrawal are also associated with increased *ChAT, Slc5a7*, and *Slc18a3* expression in the NAc (Eipper-Mains et al., 2013). Others also have reported that alcohol consumption reduces ChAT in NAc, resulting in fewer basal forebrain cholinergic neurons (Jamal et al., 2007; Coleman et al., 2011; Pereira et al., 2014). Thus, the cholinergic neuron function in the NAc deserves further investigation with regard to its involvement in female drinking. Another significant finding is the down-regulation of *Ngfr* and *Lhx8*, resulting in alterations of *Ntrk1*, which might regulate downstream MAPK signaling and Wnt (Figure 8).

We speculate that a potential mechanism for sex-dependent differences in gene expression is that promoter variants between the iP and ISCS-B lines alter the binding affinity for *trans-acting* factors, such as estrogen receptor (Figure 6), resulting in different levels of mRNA expression between female ISCS-B and iP controls. Because two interrelated pathways were found to be enriched for genes associated with female alcohol consumption or response to estrogen regulation (Figure 7); therefore, we further speculate that upregulation of *Adcyap1r1* may affect adenylyl cyclase signaling resulting in neuron protection and downregulation of *Ntrk* and *Ngf* signaling resulting in reduced apoptosis. These data suggest that AC and cholinergic function may play important roles in female alcohol consumption. Additionally, BDNF, C-jun and estradiol are all significant upstream regulators that affect DEG expression and are functionally important in these pathways. Together, these findings demonstrate a potential role for estrogen in the upregulation of *Adcyap1r1* expression that is associated with a sex-specific QTL for alcohol consumption on rat chromosome 4.

## Author Contributions

TL along with JPS, JLR, and WY designed the research and wrote the manuscript. JPS, TG, KEW, and PJB performed the animal model development, alcohol consumption test, and molecular analysis in the research. WY along with BQ, HG, and LZ performed cloning and transient transfection. DKG and ZL executed the RNA-seq. YZ along with TL, WY, and JPS analyzed the data. All authors have read and given final approval for the manuscript.

## Conflict of Interest Statement

The authors declare that the research was conducted in the absence of any commercial or financial relationships that could be construed as a potential conflict of interest.

## Footnotes

1 https://www.ncbi.nlm.nih.gov/geo

2 https://www.qiagenbioinformatics.com/products/ingenuity-pathway-analysis/

3 www.reactome.org

4 http://www.genomatix.de/

## Supplementary Material

FIGURE S1 Female ISCS-B rats demonstrated less consumption than iP and ISCSB-H. Males demonstrated no differences between strains. Female heterozygotes (ISCSB-H) showed a similar level of alcohol consumption when compared to iP controls.

FIGURE S2 Female and male ISCS-B rats exhibited similar consumption of either saccharin or quinine indicating no taste difference between sexes.

FIGURE S3 Differentially expressed genes were plotted by gene expression and fold change. The top five Ingenuity Pathway Analysis networks, diseases and bio functions are listed.

TABLE S1 RNA-seq reads of each sample.

TABLE S2 Gene expression in the nucleus accumbens of both male and female rats using RT-qPCR.

TABLE S3 Differentially expressed gene list.

TABLE S4 Significant top regulator effect networks.

TABLE S5 List of primer sequences.
