# Supplementary figures and images for: Estrogen-Dependent Upregulation of Adcyap1r1 Expression in Nucleus Accumbens Is Associated With Genetic Predisposition of Sex-Specific QTL for Alcohol Consumption on Rat Chromosome 4
Source: Front Genet. 2018 Dec 4;9:513. doi: 10.3389/fgene.2018.00513 (PMC6288178; doi:10.3389/fgene.2018.00513)

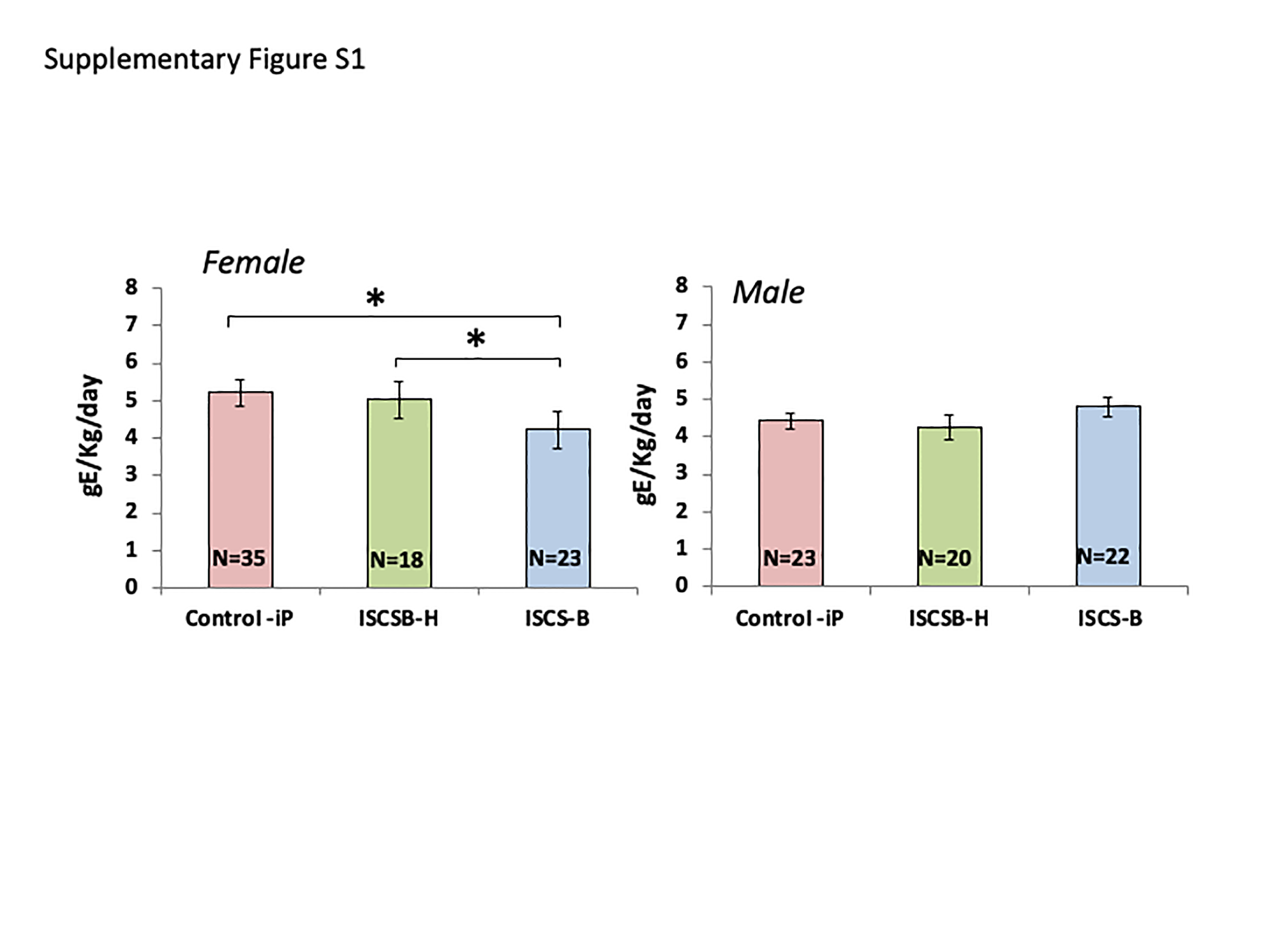

Supplement: FIGURE S1 — Female ISCS-B rats demonstrated less consumption than iP and ISCSB-H. Males demonstrated no differences between strains. Female heterozygotes (ISCSB-H) showed a similar level of alcohol consumption when compared to iP controls. [file Image_1.tif]

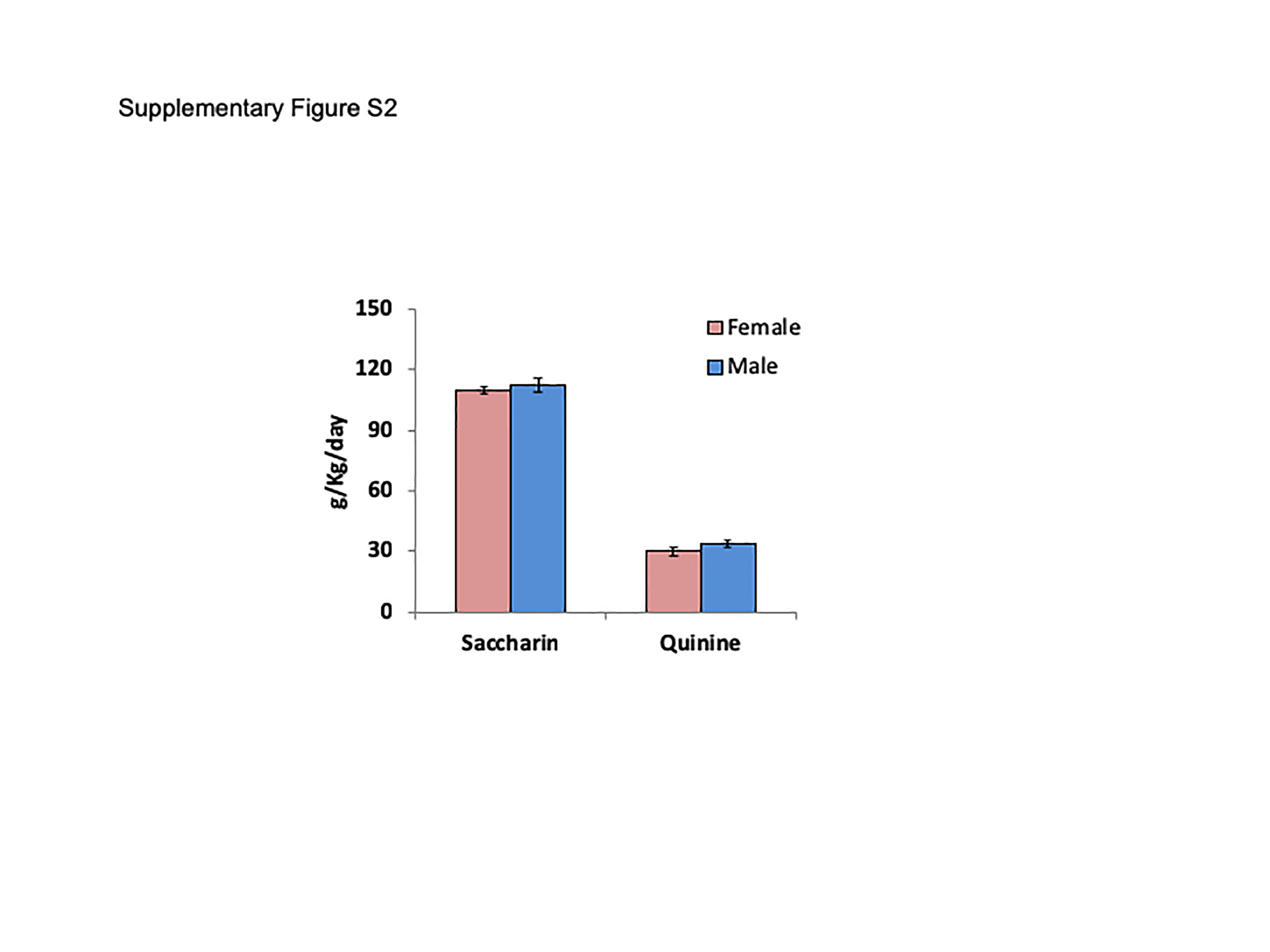

Supplement: FIGURE S2 — Female and male ISCS-B rats exhibited similar consumption of either saccharin or quinine indicating no taste difference between sexes. [file Image_2.tif]

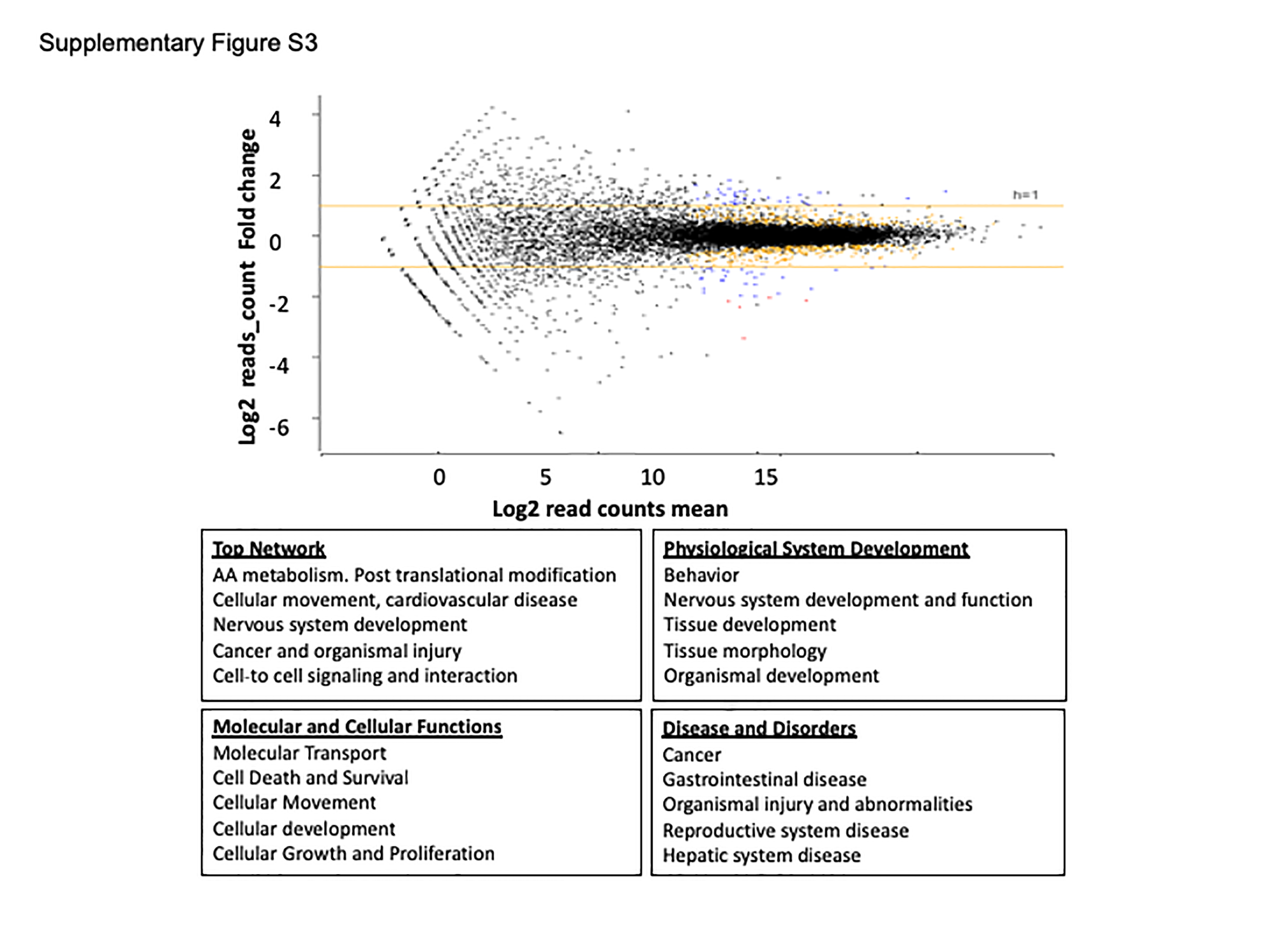

Supplement: FIGURE S3 — Differentially expressed genes were plotted by gene expression and fold change. The top five Ingenuity Pathway Analysis networks, diseases and bio functions are listed. [file Image_3.tif]
