# Supplementary material for: Estrogen-Dependent Upregulation of Adcyap1r1 Expression in Nucleus Accumbens Is Associated With Genetic Predisposition of Sex-Specific QTL for Alcohol Consumption on Rat Chromosome 4
Source: Front Genet. 2018 Dec 4;9:513. doi: 10.3389/fgene.2018.00513 (PMC6288178; doi:10.3389/fgene.2018.00513)
Supplement: TABLE S1 — RNA-seq reads of each sample. [file Table_1.pdf]

Sample	iP-F2	iP-F5	iP-F6	ISCS-B-F9	ISCS-B-F10	ISCS-B-F11
Filtered paired reads	29,973,030	34,661,705	44,114,178	49,441,621	48,486,251	46,258,263
Left mapped reads	29,971,923	34,660,505	44,112,528	49,439,481	48,484,493	46,258,263
Left mapped reads (%)	100%	100%	100%	100%	100%	100%
Right mapped reads	29,947,938	34,633,262	44,085,777	49,400,296	48,454,937	46,228,550
Right mapped reads (%)	99.92%	99.92%	99.94%	99.92%	99.94%	99.94%

Supplementary Table S1. RNA-seq reads of each sample.

Female

Gene Symbol	ISCS9 (mean)	Variance	iP (mean)	Variance	Fold change: ISCSB/iP	p-value (one-tail)
Adcyap1r1	1.927	0.063	1.495	0.238	1.289	0.001
Adcy7	0.54	0.01	0.57	0.01	0.946	0.183
Slc5a7	1.06	0.18	1.24	0.15	0.851	0.092
Tacr1	1.278	0.218	1.516	0.116	0.843	0.046
Slc18a3	0.906	0.108	1.262	0.277	0.718	0.011
lhx8	2.15	1.07	3.16	1.19	0.681	0.004
Chat	2.26	1.01	3.52	2.61	0.642	0.004
Ngfr	*0.268*	0.009	*0.438*	0.046	0.611	0.003
Nap1L5	1.27	0.02	1.01	0.03	1.252	0.000
Snca	1.78	0.13	1.09	0.03	1.631	0.000
Ppm1K	1.45	0.01	1.04	0.02	1.393	0.000
Aqp1	0.85	0.29	1.25	0.64	0.681	0.044

Male

	ISCS9 (mean)	Variance	iP (mean)	Variance	ld change: ISCSB/	p-value (one-tail)
Adcyap1r1	1.863	0.071	1.980	0.213	0.941	0.199
Adcy7	0.557	0.013	0.734	0.057	0.759	0.009
Slc5a7	1.165	0.446	3.408	6.201	0.342	0.002
Tacr1	1.214	0.197	2.515	0.905	0.483	0.000
Slc18a3	1.083	0.377	1.988	1.641	0.545	0.011
Lhx8	2.74	4.05	7.69	29.62	0.355	0.002
Chat	2.97	3.10	7.29	26.03	0.407	0.004
Ngfr	*0.25*	0.01	*1.11*	1.32	0.227	0.006
Nap1L5	1.01	0.06	1.05	0.12	0.965	0.356
Snca	1.31	0.03	1.04	0.07	1.263	0.001
PpmiK	1.47	0.03	1.08	0.12	1.371	0.000
Aqp1	2.38	3.79	1.33	1.38	1.786	0.030

Supplementary. Table S2. Gene expression in the Nac of both male and female rats using RT-qPCR

Ensemb ID	Gene symbol	Full name	Chromosome	BaseMean_iP	BaseMean_ISCS-B	FC_2	log2FC_2	pval	padj
ENSRNOG00000010597	Slc5a7	solute carrier family 5 (sodium/choline cotransporter); member 7	chr9	1907.52	449.09	0.235	-2.09	0.00	0.000
ENSRNOG00000025012	Chat	choline O-acetyltransferase	chr16	944.03	235.43	0.249	-2.00	0.00	0.000
ENSRNOG00000019447	Ecel1	endothelin converting enzyme-like 1	chr9	2243.20	674.62	0.301	-1.73	0.00	0.000
ENSRNOG00000026091	Slc10a4	solute carrier family 10; member 4	chr14	719.90	180.36	0.251	-2.00	0.00	0.000
ENSRNOG00000062141	Slc18a3	solute carrier family 18 member A3	chr16	394.56	88.07	0.223	-2.16	0.00	0.000
ENSRNOG00000013953	Ntrk1	neurotrophic tyrosine kinase; receptor; type 1	chr2	502.74	129.07	0.257	-1.96	0.00	0.000
ENSRNOG00000015835	Cacna2d2	calcium channel; voltage-dependent; alpha 2/delta subunit 2	chr8	3379.89	1580.50	0.468	-1.10	0.00	0.000
ENSRNOG00000005392	Ngfr	nerve growth factor receptor	chr10	615.47	61.58	0.100	-3.32	0.00	0.000
ENSRNOG00000050841	Ache	acetylcholinesterase	chr12	2748.55	1127.17	0.410	-1.29	0.00	0.000
ENSRNOG00000008176	Nppa	natriuretic peptide A	chr5	171.07	423.32	2.475	1.31	0.00	0.000
ENSRNOG00000028348	Lhx8	LIM homeobox 8	chr2	511.42	95.09	0.186	-2.43	0.00	0.000
ENSRNOG00000021084	Mpeg1	macrophage expressed 1	chr1	1237.10	565.67	0.457	-1.13	0.00	0.000
ENSRNOG00000005996	Lhx6	LIM homeobox 6	chr3	1090.45	427.30	0.392	-1.35	0.00	0.000
ENSRNOG00000007581	Slc17a8	solute carrier family 17 (vesicular glutamate transporter); member 8	chr7	277.44	110.02	0.397	-1.33	0.00	0.000
ENSRNOG00000005853	Tacr1	tachykinin receptor 1	chr4	421.33	194.06	0.461	-1.12	0.00	0.000
ENSRNOG00000031397	Stk32b	serine/threonine kinase 32B	chr14	113.78	31.89	0.280	-1.84	0.00	0.000
ENSRNOG00000003066	Wnt9a	wingless-type MMTV integration site family; member 9A	chr10	163.63	353.69	2.162	1.11	0.00	0.000
ENSRNOG00000059714	Hsp90aa1	heat shock protein 90; alpha (cytosolic); class A member 1	chr6	397.27	193.89	0.488	-1.03	0.00	0.000
ENSRNOG00000028708	Ntsr1	neurotensin receptor 1	chr3	175.63	53.57	0.305	-1.71	0.00	0.000
ENSRNOG00000008855	Gjb2	gap junction protein; beta 2	chr15	243.93	687.01	2.816	1.49	0.00	0.000
ENSRNOG00000006120	Shh	sonic hedgehog	chr4	127.48	41.34	0.324	-1.62	0.00	0.000
ENSRNOG00000021745	Bhlhe22	basic helix-loop-helix family; member e22	chr2	175.92	477.28	2.713	1.44	0.00	0.000
ENSRNOG00000008697	Nov	nephroblastoma overexpressed	chr7	652.83	1675.98	2.567	1.36	0.00	0.000
ENSRNOG00000016057	Kcnj13	potassium channel; inwardly rectifying subfamily J; member 13	chr9	47.25	133.95	2.835	1.50	0.00	0.000
ENSRNOG00000021063	Grin2d	glutamate receptor; ionotropic; N-methyl D-aspartate 2D	chr1	551.57	233.09	0.423	-1.24	0.00	0.000
ENSRNOG00000003183	Fmod	fibromodulin	chr13	455.54	991.71	2.177	1.12	0.00	0.000
ENSRNOG00000046863	C1ql2	complement component 1; q subcomponent-like 2	chr13	141.25	52.60	0.372	-1.43	0.00	0.000
ENSRNOG00000053384	Bmp7	bone morphogenetic protein 7	chr3	155.33	396.83	2.555	1.35	0.00	0.000
ENSRNOG00000004372	Cbln4	cerebellin 4 precursor	chr3	323.56	130.42	0.403	-1.31	0.00	0.000
ENSRNOG00000005580	Itgb4	integrin; beta 4	chr10	221.95	498.72	2.247	1.17	0.00	0.000
ENSRNOG00000015957	F13a1	coagulation factor XIII; A1 polypeptide	chr17	57.84	143.34	2.478	1.31	0.00	0.000
ENSRNOG00000027220	Pcdhgb8	protocadherin gamma subfamily B; 8	chr18	218.08	104.31	0.478	-1.06	0.00	0.000

ENSRNOG00000020620	Ppp1r32	protein phosphatase 1; regulatory subunit 32	chr1	85.04	26.50	0.312	-1.68	0.00	0.000
ENSRNOG00000055049	Aldh1a2	aldehyde dehydrogenase 1 family; member A2	chr8	330.43	792.82	2.399	1.26	0.00	0.000
ENSRNOG00000004210	Osr1	odd-skipped related transciption factor 1	chr6	51.57	151.03	2.928	1.55	0.00	0.000
ENSRNOG00000019495	Gbx2	gastrulation brain homeobox 2	chr9	97.20	35.79	0.368	-1.44	0.00	0.000
ENSRNOG00000057092	Slfn3	schlafen 3	chr10	40.56	106.64	2.629	1.39	0.00	0.000
ENSRNOG00000026110	Scml4	sex comb on midleg-like 4 (Drosophila)	chr20	194.72	94.20	0.484	-1.05	0.00	0.000
ENSRNOG00000016957	Igfbp2	insulin-like growth factor binding protein 2	chr9	716.69	1576.29	2.199	1.14	0.00	0.000
ENSRNOG00000018715	Clec10a	C-type lectin domain family 10; member A	chr10	34.74	95.00	2.735	1.45	0.00	0.000
ENSRNOG00000032844	RT1-Da	RT1 class II; locus Da	chr20	42.83	124.97	2.918	1.55	0.00	0.000
ENSRNOG00000016085	Mpzl2	myelin protein zero-like 2	chr8	49.78	191.67	3.851	1.95	0.00	0.000
ENSRNOG00000004614	Lypd6b	LY6/PLAUR domain containing 6B	chr3	251.59	112.77	0.448	-1.16	0.00	0.000
ENSRNOG00000020369	Igf2	insulin-like growth factor 2	chr1	1486.36	2997.30	2.017	1.01	0.00	0.000
ENSRNOG00000056714	Sla	src-like adaptor	chr7	210.11	458.74	2.183	1.13	0.00	0.000
ENSRNOG00000015259	Pth2r	parathyroid hormone 2 receptor	chr9	65.84	20.82	0.316	-1.66	0.00	0.000
ENSRNOG00000015550	Ptgds	prostaglandin D2 synthase (brain)	chr3	13019.93	35901.42	2.757	1.46	0.00	0.000
ENSRNOG00000012727	Trpc7	transient receptor potential cation channel; subfamily C; member 7	chr17	237.69	111.63	0.470	-1.09	0.00	0.000
ENSRNOG00000000723	RT1-CE5	RT1 class I; locus CE5	chr20	62.06	134.65	2.170	1.12	0.00	0.000
ENSRNOG00000030763	Dpp4	dipeptidylpeptidase 4	chr3	48.24	118.80	2.463	1.30	0.00	0.000
ENSRNOG00000046848	PCOLCE2	procollagen C-endopeptidase enhancer 2	chr8	31.42	85.66	2.726	1.45	0.00	0.000
ENSRNOG00000004179	Nts	neurotensin	chr7	627.17	240.83	0.384	-1.38	0.00	0.000
ENSRNOG00000015071	Zim1	zinc finger; imprinted 1	chr1	237.23	65.66	0.277	-1.85	0.00	0.000
ENSRNOG00000019141	Ch25h	cholesterol 25-hydroxylase	chr1	51.27	121.82	2.376	1.25	0.00	0.000
ENSRNOG00000013369	Gbx1	gastrulation brain homeobox 1	chr4	52.01	15.92	0.306	-1.71	0.00	0.000
ENSRNOG00000030530	Gzmm	granzyme M (lymphocyte met-ase 1)	chr7	34.83	87.85	2.522	1.33	0.00	0.000
ENSRNOG00000018215	Slc22a6	solute carrier family 22 (organic anion transporter); member 6	chr1	193.48	524.39	2.710	1.44	0.00	0.000
ENSRNOG00000017409	Wnt6	wingless-type MMTV integration site family; member 6	chr9	59.52	188.51	3.167	1.66	0.00	0.000
ENSRNOG00000011648	Aqp1	aquaporin 1	chr4	53.34	178.68	3.350	1.74	0.00	0.000
ENSRNOG00000015668	Ccl19	chemokine (C-C motif) ligand 19	chr5	39.47	94.96	2.406	1.27	0.00	0.000
ENSRNOG00000049213	Npy2r	neuropeptide Y receptor Y2	chr2	126.61	56.71	0.448	-1.16	0.00	0.000
ENSRNOG00000018735	Cd74	Cd74 molecule; major histocompatibility complex; class II	chr18	128.83	375.80	2.917	1.54	0.00	0.000
ENSRNOG00000055705	Htr7	5-hydroxytryptamine (serotonin) receptor 7; adenylate cyclase-coupled	chr1	112.45	53.51	0.476	-1.07	0.00	0.000
ENSRNOG00000003172	Serpinf1	serpin peptidase inhibitor; clade F (alpha-2 antiplasmin; pigment	chr10	159.55	421.71	2.643	1.40	0.00	0.001
ENSRNOG00000026907	Zbtb37	zinc finger and BTB domain containing 37	chr13	61.34	22.85	0.373	-1.42	0.00	0.001

ENSRNOG00000019118	Slc13a3	solute carrier family 13 (sodium-dependent dicarboxylate transporter);	chr3	808.48	1679.85	2.078	1.06	0.00	0.001
ENSRNOG00000027392	Ccdc187	coiled-coil domain containing 187	chr3	185.69	81.53	0.439	-1.19	0.00	0.001
ENSRNOG00000018251	Mrc1	mannose receptor; C type 1	chr17	153.15	311.13	2.031	1.02	0.00	0.001
ENSRNOG00000025670	Shisa3	shisa family member 3	chr14	110.59	384.35	3.475	1.80	0.00	0.001
ENSRNOG00000027935	Lrrc34	leucine rich repeat containing 34	chr2	60.78	19.72	0.324	-1.62	0.00	0.001
ENSRNOG00000008644	Nkx2-1	NK2 homeobox 1	chr6	145.37	47.39	0.326	-1.62	0.00	0.001
N/A	LOC310926	hypothetical protein LOC310926	chr1	47.59	225.83	4.746	2.25	0.00	0.001
ENSRNOG00000012995	Gpr165	G protein-coupled receptor 165	chrX	1103.53	295.73	0.268	-1.90	0.00	0.001
ENSRNOG00000013426	Mrgprf	MAS-related GPR; member F	chr1	33.28	84.71	2.545	1.35	0.00	0.001
ENSRNOG00000011642	Tfap2d	transcription factor AP-2 delta	chr9	25.23	64.33	2.550	1.35	0.00	0.002
ENSRNOG00000046972	Chrm2	cholinergic receptor; muscarinic 2	chr4	274.75	132.94	0.484	-1.05	0.00	0.002
ENSRNOG00000017459	C1ql3	complement component 1; q subcomponent-like 3	chr17	211.42	427.34	2.021	1.02	0.00	0.002
ENSRNOG00000010128	Slc27a2	solute carrier family 27 (fatty acid transporter); member 2	chr3	170.64	55.36	0.324	-1.62	0.00	0.002
ENSRNOG00000014776	Adcy7	adenylate cyclase 7	chr19	345.31	152.71	0.442	-1.18	0.00	0.002
ENSRNOG00000012448	Chrnb3	cholinergic receptor; nicotinic; beta 3 (neuronal)	chr16	23.14	59.53	2.573	1.36	0.00	0.002
ENSRNOG00000023576	RGD1305645	similar to RIKEN cDNA 1500015O10	chr9	51.08	140.24	2.746	1.46	0.00	0.002
ENSRNOG00000048924	Islr	immunoglobulin superfamily containing leucine-rich repeat	chr8	160.70	377.94	2.352	1.23	0.00	0.003
ENSRNOG00000039086	Ccdc153	coiled-coil domain containing 153	chr8	81.94	38.58	0.471	-1.09	0.00	0.003
ENSRNOG00000038600	Dnaaf3	dynein; axonemal; assembly factor 3	chr1	98.84	48.18	0.487	-1.04	0.00	0.003
ENSRNOG00000012608	Tmem212	transmembrane protein 212	chr2	35.00	1.44	0.041	-4.60	0.00	0.003
ENSRNOG00000011821	S100a4	S100 calcium-binding protein A4	chr2	47.94	112.81	2.353	1.23	0.00	0.003
ENSRNOG00000018087	Vim	vimentin	chr17	1688.49	3433.83	2.034	1.02	0.00	0.003
ENSRNOG00000031100	Klhl1	kelch-like family member 1	chr15	130.30	32.82	0.252	-1.99	0.00	0.003
ENSRNOG00000011184	Slc13a4	solute carrier family 13 (sodium/sulfate symporter); member 4	chr4	517.88	1270.80	2.454	1.30	0.00	0.004
ENSRNOG00000025691	Pla2g7	phospholipase A2; group VII (platelet-activating factor acetylhydrolase;	chr9	254.10	565.86	2.227	1.16	0.00	0.004
N/A	Sertm2	serine rich and transmembrane domain containing 2	chrX	126.16	56.28	0.446	-1.16	0.00	0.004
ENSRNOG00000011823	Tfap2b	transcription factor AP-2 beta	chr9	38.77	108.85	2.808	1.49	0.00	0.004
ENSRNOG00000011251	Hcrtr2	hypocretin (orexin) receptor 2	chr8	89.96	37.48	0.417	-1.26	0.00	0.005
ENSRNOG00000060867	Rln1	relaxin 1	chr1	18.08	48.48	2.681	1.42	0.00	0.006
ENSRNOG00000028041	Tnnt1	troponin T type 1 (skeletal; slow)	chr1	162.73	76.38	0.469	-1.09	0.00	0.007
ENSRNOG00000035555	Mir384	microRNA 384	chrX	18.22	2.92	0.160	-2.64	0.00	0.007
ENSRNOG00000013720	Aebp1	AE binding protein 1	chr14	891.93	1813.33	2.033	1.02	0.00	0.007
ENSRNOG00000022957	Ctxn3	cortexin 3	chr18	293.69	135.06	0.460	-1.12	0.00	0.007

ENSRNOG00000013681	Kcns1	potassium voltage-gated channel; modifier subfamily S; member 1	chr3	39.16	79.33	2.026	1.02	0.00	0.008
ENSRNOG00000051684	St8sia6	ST8 alpha-N-acetyl-neuraminide alpha-2;8-sialyltransferase 6	chr17	24.96	6.40	0.256	-1.96	0.00	0.008
ENSRNOG00000008492	Ccdc19	cilia and flagella associated protein 45 (Cfap45)	chr13	46.23	17.71	0.383	-1.38	0.00	0.009
ENSRNOG00000002120	Spata18	spermatogenesis associated 18	chr14	47.73	5.79	0.121	-3.04	0.00	0.009
ENSRNOG00000013768	Defb1	defensin beta 1	chr16	21.26	52.43	2.466	1.30	0.00	0.009
N/A	Tnxa-ps1	tenascin XA, pseudogene 1	chr20	36.39	76.25	2.096	1.07	0.00	0.010
ENSRNOG00000006639	Scn9a	sodium channel; voltage-gated; type IX; alpha subunit	chr3	706.54	335.16	0.474	-1.08	0.00	0.010
ENSRNOG00000040287	Cyp1b1	cytochrome P450; family 1; subfamily b; polypeptide 1	chr6	31.89	87.04	2.730	1.45	0.00	0.010
N/A	Gstm2	glutathione S-transferase mu 2	chr2	50.33	156.58	3.111	1.64	0.00	0.011
ENSRNOG00000018346	Agtr1a	angiotensin II receptor; type 1a	chr17	31.96	67.92	2.125	1.09	0.00	0.012
ENSRNOG00000005781	Wnt16	wingless-type MMTV integration site family; member 16	chr4	52.20	107.93	2.068	1.05	0.00	0.012
ENSRNOG00000019358	Esr1	estrogen receptor 1	chr1	63.93	15.29	0.239	-2.06	0.00	0.013
ENSRNOG00000052564	Gpx3	glutathione peroxidase 3	chr10	634.95	208.23	0.328	-1.61	0.00	0.014
ENSRNOG00000031211	Acsm5	acyl-CoA synthetase medium-chain family member 5	chr1	65.09	131.20	2.016	1.01	0.00	0.014
ENSRNOG00000047098	Hbb	beta-globin	chr1	55.03	198.24	3.602	1.85	0.00	0.014
ENSRNOG00000000451	RT1-Ba	RT1 class II; locus Ba	chr20	34.91	71.82	2.057	1.04	0.00	0.015
ENSRNOG00000061182	Gabre	gamma-aminobutyric acid (GABA) A receptor; epsilon	chrX	117.35	10.41	0.089	-3.49	0.00	0.017
ENSRNOG00000018316	Grap2	GRB2-related adaptor protein 2	chr7	17.10	42.76	2.500	1.32	0.00	0.017
ENSRNOG00000010253	Cd163	CD163 molecule	chr4	54.68	114.77	2.099	1.07	0.00	0.017
ENSRNOG00000002862	Clcn5	chloride channel; voltage-sensitive 5	chrX	207.26	81.64	0.394	-1.34	0.00	0.018
ENSRNOG00000030021	Ccl6	chemokine (C-C motif) ligand 6	chr10	22.10	51.16	2.315	1.21	0.00	0.018
ENSRNOG00000059852	Rn5-8s	5.8S ribosomal RNA	chr14	2.95	21.96	7.453	2.90	0.00	0.019
ENSRNOG00000021225	Oxt	oxytocin/neurophysin 1 prepropeptide	chr3	446.57	1.17	0.003	-8.58	0.00	0.019
ENSRNOG00000041468	Mir1188	microRNA 1188	chr6	56.85	26.72	0.470	-1.09	0.00	0.020
ENSRNOG00000010362	Anxa2	annexin A2	chr8	215.47	450.40	2.090	1.06	0.00	0.022
ENSRNOG00000042139	Clec4a1	C-type lectin domain family 4; member A1	chr4	21.36	47.76	2.237	1.16	0.00	0.022
ENSRNOG00000009372	Tacr3	tachykinin receptor 3	chr2	132.52	58.11	0.439	-1.19	0.00	0.023
ENSRNOG00000020151	Cdh1	cadherin 1	chr19	97.64	284.01	2.909	1.54	0.00	0.023
ENSRNOG00000013838	Hcrtr1	hypocretin (orexin) receptor 1	chr5	51.93	23.47	0.452	-1.15	0.00	0.024
ENSRNOG00000019890	Folr2	folate receptor 2 (fetal)	chr1	10.67	31.06	2.911	1.54	0.00	0.024
N/A	Capns2	calpain, small subunit 2	chr19	50.67	23.19	0.458	-1.13	0.00	0.025
ENSRNOG00000028548	Ccl9	chemokine (C-C motif) ligand 9	chr10	9.81	33.78	3.442	1.78	0.00	0.025
ENSRNOG00000028015	Pf4	platelet factor 4	chr14	17.90	44.35	2.478	1.31	0.00	0.026

ENSRNOG00000023664	Lepr	leptin receptor	chr5	224.22	460.22	2.052	1.04	0.00	0.026
ENSRNOG00000012301	Cfap161	cilia and flagella associated protein 161	chr1	38.19	15.39	0.403	-1.31	0.00	0.029
ENSRNOG00000033734	Tnnt2	troponin T type 2 (cardiac)	chr13	132.76	334.28	2.518	1.33	0.00	0.029
ENSRNOG00000027658	Gpr101	G protein-coupled receptor 101	chrX	733.37	359.77	0.491	-1.03	0.00	0.030
ENSRNOG00000012450	Dynlrb2	dynein light chain roadblock-type 2	chr19	27.75	8.41	0.303	-1.72	0.00	0.031
ENSRNOG00000018445	Agt	angiotensinogen (serpin peptidase inhibitor; clade A; member 8)	chr19	6663.91	3223.93	0.484	-1.05	0.00	0.032
ENSRNOG00000007765	Frzb	frizzled-related protein	chr3	173.78	390.25	2.246	1.17	0.00	0.033
ENSRNOG00000022256	Cxcl10	chemokine (C-X-C motif) ligand 10	chr14	7.60	24.64	3.245	1.70	0.00	0.034
ENSRNOG00000020650	Slc17a7	solute carrier family 17 (vesicular glutamate transporter); member 7	chr1	6614.15	15565.80	2.353	1.23	0.00	0.036
ENSRNOG00000037060	Odf3b	outer dense fiber of sperm tails 3B	chr7	56.24	27.66	0.492	-1.02	0.00	0.036
ENSRNOG00000061299	LOC100134871	beta globin minor gene	chr1	200.83	761.83	3.793	1.92	0.00	0.037
ENSRNOG00000026150	Zar1	zygote arrest 1	chr14	43.63	19.11	0.438	-1.19	0.00	0.038
ENSRNOG00000007457	Serping1	serpin peptidase inhibitor; clade G (C1 inhibitor); member 1	chr3	218.89	470.52	2.150	1.10	0.00	0.041
ENSRNOG00000003357	Col3a1	collagen; type III; alpha 1	chr9	564.80	1146.39	2.030	1.02	0.00	0.044
ENSRNOG00000018693	Asgr1	asialoglycoprotein receptor 1	chr10	21.21	45.89	2.164	1.11	0.00	0.045
ENSRNOG00000014893	Wdr63	WD repeat domain 63	chr2	76.19	15.38	0.202	-2.31	0.00	0.045
ENSRNOG00000026661	Gpr81	G protein-coupled receptor 81	chr12	19.20	42.62	2.220	1.15	0.00	0.049
ENSRNOG00000002881	Ddr2	discoidin domain receptor tyrosine kinase 2	chr13	184.83	378.47	2.048	1.03	0.00	0.050
ENSRNOG00000018191	Oprm1	opioid receptor; mu 1	chr1	199.63	76.55	0.383	-1.38	0.00	0.053
ENSRNOG00000009197	Asb4	ankyrin repeat and SOCS box-containing 4	chr4	51.62	3.75	0.073	-3.78	0.00	0.054
ENSRNOG00000058105	Hbb-b1	hemoglobin, beta adult major chain	chr1	245.57	759.81	3.094	1.63	0.00	0.055
ENSRNOG00000017416	Ppic	peptidylprolyl isomerase C	chr18	47.06	107.02	2.274	1.19	0.00	0.056
ENSRNOG00000047763	Mir3547	microRNA 3547	chr10	102.89	35.15	0.342	-1.55	0.00	0.058
ENSRNOG00000019584	Dlk1	delta-like 1 homolog (Drosophila)	chr6	477.30	165.13	0.346	-1.53	0.00	0.070
ENSRNOG00000059469	Otx1	orthodenticle homeobox 1	chr14	23.65	48.43	2.047	1.03	0.00	0.071
ENSRNOG00000012843	Aspg	asparaginase	chr6	36.16	112.50	3.111	1.64	0.00	0.074
ENSRNOG00000016654	Galr1	galanin receptor 1	chr18	56.93	21.77	0.382	-1.39	0.00	0.075
ENSRNOG00000018290	Alx3	ALX homeobox 3	chr2	22.32	52.26	2.341	1.23	0.00	0.078
ENSRNOG00000016581	Serpinb1a	serine (or cysteine) proteinase inhibitor; clade B; member 1a	chr17	52.70	118.43	2.247	1.17	0.00	0.078
ENSRNOG00000016678	Angptl2	angiopoietin-like 2	chr3	70.74	158.80	2.245	1.17	0.01	0.083
ENSRNOG00000062013	Adprhl1	ADP-ribosylhydrolase like 1	chr16	14.84	32.81	2.211	1.14	0.01	0.086
ENSRNOG00000002217	Plac8	placenta-specific 8	chr14	9.02	25.18	2.793	1.48	0.01	0.087
ENSRNOG00000028417	Neurod2	neuronal differentiation 2	chr10	179.69	362.67	2.018	1.01	0.01	0.087

ENSRNOG00000004712	Angptl1	angiopoietin-like 1	chr13	23.35	52.25	2.237	1.16	0.01	0.089
ENSRNOG00000011568	Rspo3	R-spondin 3	chr1	70.86	151.03	2.131	1.09	0.01	0.094
ENSRNOG00000002385	Prg4	proteoglycan 4	chr13	131.08	284.57	2.171	1.12	0.01	0.095
ENSRNOG00000006146	Trim54	tripartite motif-containing 54	chr6	221.62	523.17	2.361	1.24	0.01	0.095
ENSRNOG00000011101	Twist1	twist family bHLH transcription factor 1	chr6	21.61	50.09	2.318	1.21	0.01	0.102
ENSRNOG00000019445	Msln	mesothelin	chr10	18.75	73.88	3.939	1.98	0.01	0.104
ENSRNOG00000053402	Gabrq	gamma-aminobutyric acid (GABA) A receptor; theta	chrX	79.36	11.11	0.140	-2.84	0.01	0.111
ENSRNOG00000005049	Tbr1	T-box; brain; 1	chr3	340.47	755.02	2.218	1.15	0.01	0.112
ENSRNOG00000049792	Glra3	glycine receptor; alpha 3	chr16	29.31	12.74	0.435	-1.20	0.01	0.117
ENSRNOG00000023991	Rab20	RAB20; member RAS oncogene family	chr16	15.18	33.24	2.190	1.13	0.01	0.118
ENSRNOG00000058560	Col2a1	collagen; type II; alpha 1	chr7	159.84	53.87	0.337	-1.57	0.01	0.122
ENSRNOG00000009005	Slco2a1	solute carrier organic anion transporter family; member 2a1	chr8	54.16	141.26	2.608	1.38	0.01	0.122
ENSRNOG00000047446	Foxc2	forkhead box C2	chr19	26.98	76.31	2.829	1.50	0.01	0.125
ENSRNOG00000010053	Calcr	calcitonin receptor	chr4	714.88	150.72	0.211	-2.25	0.01	0.130
ENSRNOG00000015599	Mall	mal; T-cell differentiation protein-like	chr3	19.45	40.46	2.080	1.06	0.01	0.136
ENSRNOG00000024172	T2	brachyury 2	chr1	52.76	24.64	0.467	-1.10	0.01	0.142
ENSRNOG00000010699	Trim43a	tripartite motif-containing 43A	chr8	29.84	13.86	0.465	-1.11	0.01	0.150
ENSRNOG00000039107	Mfrp	membrane frizzled-related protein	chr8	52.76	146.83	2.783	1.48	0.01	0.150
ENSRNOG00000005930	Nnmt	nicotinamide N-methyltransferase	chr8	14.25	29.90	2.098	1.07	0.01	0.151
ENSRNOG00000007027	Hgf	hepatocyte growth factor	chr4	44.24	89.90	2.032	1.02	0.02	0.166
ENSRNOG00000008394	Prg2	proteoglycan 2	chr3	8.53	20.60	2.415	1.27	0.02	0.179
ENSRNOG00000006090	Ucn	urocortin	chr6	6.04	16.31	2.702	1.43	0.02	0.179
ENSRNOG00000006204	Slc30a3	solute carrier family 30 (zinc transporter); member 3	chr6	825.69	1776.57	2.152	1.11	0.02	0.182
ENSRNOG00000001959	Mx1	myxovirus (influenza virus) resistance 1	chr11	21.67	78.82	3.638	1.86	0.02	0.186
ENSRNOG00000047917	Plscr5	phospholipid scramblase family; member 5	chr8	20.45	9.06	0.443	-1.17	0.02	0.189
ENSRNOG00000037149	Bcl2l15	BCL2-like 15	chr2	23.97	11.00	0.459	-1.12	0.02	0.191
ENSRNOG00000011160	Sv2b	synaptic vesicle glycoprotein 2b	chr1	4074.95	8335.78	2.046	1.03	0.02	0.195
ENSRNOG00000017783	Sfrp1	secreted frizzled-related protein 1	chr16	388.47	867.27	2.233	1.16	0.02	0.195
ENSRNOG00000005048	Trhr	thyrotropin releasing hormone receptor	chr7	167.52	73.43	0.438	-1.19	0.02	0.201
ENSRNOG00000032063	Gfral	GDNF family receptor alpha like	chr8	30.65	66.42	2.167	1.12	0.02	0.204
ENSRNOG00000036267	Mir341	microRNA 341	chr6	23.22	10.75	0.463	-1.11	0.02	0.204
ENSRNOG00000026055	Neurod6	neuronal differentiation 6	chr4	209.83	471.69	2.248	1.17	0.02	0.204
ENSRNOG00000021323	Car13	carbonic anhydrase 13	chr2	41.87	90.18	2.154	1.11	0.03	0.214

N/A	Hist3h3	histone cluster 3, H3	chr10	18.49	7.54	0.408	-1.29	0.03	0.227
ENSRNOG00000016275	Ttr	transthyretin	chr18	7.60	40.13	5.284	2.40	0.03	0.227
ENSRNOG00000045989	Hba-a1	hemoglobin alpha; adult chain 1	chr10	38.67	123.04	3.182	1.67	0.03	0.234
ENSRNOG00000021513	Rtn4rl2	reticulon 4 receptor-like 2	chr3	127.60	297.42	2.331	1.22	0.03	0.239
ENSRNOG00000003620	Fmo3	flavin containing monooxygenase 3	chr13	18.03	44.58	2.472	1.31	0.03	0.244
ENSRNOG00000058827	Six2	SIX homeobox 2	chr6	15.94	42.57	2.671	1.42	0.03	0.245
ENSRNOG00000003959	Rgs18	regulator of G-protein signaling 18	chr13	10.77	24.83	2.306	1.21	0.03	0.245
ENSRNOG00000017672	Akr1c14	aldo-keto reductase family 1; member C14	chr17	85.10	197.81	2.324	1.22	0.03	0.249
N/A	RT1-CE16	RT1 class I; locus CE16	chr20	115.61	30.14	0.261	-1.94	0.04	0.275
ENSRNOG00000009434	RGD1310507	similar to RIKEN cDNA 1300017J02	chr8	32.93	67.66	2.055	1.04	0.04	0.276
ENSRNOG00000000167	Alas2	5'-aminolevulinate synthase 2	chrX	88.95	214.99	2.417	1.27	0.04	0.284
ENSRNOG00000012827	Mlf1	myeloid leukemia factor 1	chr2	22.38	10.62	0.474	-1.08	0.04	0.286
ENSRNOG00000041300	Mir874	microRNA 874	chr17	4.51	0.58	0.129	-2.96	0.05	0.302
ENSRNOG00000015567	Slc9a2	solute carrier family 9; subfamily A (NHE2; cation proton antiporter 2);	chr9	254.20	536.38	2.110	1.08	0.05	0.303
ENSRNOG00000037687	Rspo2	R-spondin 2	chr7	31.80	79.86	2.511	1.33	0.05	0.307

Supplementary Table S3. Differentially expressed gene list.

Top Regulator Effect Networks

ID	Diseases & Functions	Consistency Score
NGF,RAF1	Concentration of dopamine,Coordination	4.707
ASCL1, estrogen receptor, NCOA1 ,NGF, Pkc(s)	Cell movement of neurons	2.345
EBI3,IL27,JAK1/2,Pkc(s),SNCA	Formation of brain,Movement Disorders	0.765
IFN alpha/beta, IFN Beta, Ifnar, IFNL1	Glucose metabolism disorder	0.655
APP	Progressive neurological disorder	0.277

Supplementary Table S4. Significant top regulator effect networks

qRT-PCR	
lc557-gapdh-f	GAAGCTCATTTCCTGGTATGACAATG
lc558-gapdh-r	GGTTTCTTACTCCTTGGAGGCCATG
lc567-snca	AAGACTATGAGCCTGAAGCCTAAG
lc568-snca	AGTGTGAAGCCACAACAATATCC
LC631-rPpm1k-f	GCTGTGACTGAGCAGGCAATTCAG
LC631-rPpm1k-r	CTGGAGGCAAAGCTTCTGCTGAA
LC745 rNap1l5-f	AAGTGAGCACAGCAGCTGACAGACTTG
LC745 rNap1l5-r	GAGTTCTGTCTTAGCTTGGGTGTTAAGGTG
LC760 rCHAT-f	CCATTGACAACCATCTTCTGGCACTG
LC760 rCHAT-r	ATCTCCATGGTTGTGGGCACCTG
LC761 rSlc5a7-f	CCATTGGAGCCTCCACAGACTGG
LC761 rSlc5a7-r	GCAGCAGAAACAGCACCAAGCCC
LC762 rSlc18a3-f	GCCATAGCTGACATCTCCTATTCTGTGG
LC762 rSlc18a3-r	AGAAGAAGGACTGGTGCGTAGAGCAG
LC763 rLhx 8-f	CGGCGAGTCATACAGGTGTGGTTTC
LC763 rLhx 8-r	CTGGGGCACGTAGGCGGAATAAG
LC764 rNgfr-f	GCTTTCAAGAGGTGGAACAGCTGC
LC764 rNgfr-r	TGCGTATGGGTCTGCTGGTCGTG
LC765 rAdcy7 -f	CTCCGAGTTGGCATAAACCACGG
LC765 rAdcy7-r	TTCCCAAGCTCTCCGGTGCTCTC
LC766 rTacr1-f	GTTGCCTCAACGACAGGTTCCGTC
LC766 rTacr1-r	TCCAGGCGGCTGACCTTGTACAC
LC767 rTacr1-3UTR-f1	GCATATTCAAGGCATGGCCTCTAGC
LC768 rTacr1-3UTR-r1	TGCCTTCTCCAGGTATGCCGATG
LC854 rAqp1-f	GACCAGAGCCTGGACAATCTGAAG
LC855 rAqp1-r	CCAGAGAGCTAAATGCAAGCCC
Adcyap1r1 promoter cloning	
LC772 rAdcyap1r1Hind3R	TTACAAGCTTCGTCTGCTGTCGCCCACCCCG
LC773 rAdcyap1r1KpnF	ATAAGGTACCGGCTGTGAGGCAATCGGAAGAC
LC774 rAdcyap1r1_R	CCTGGAGACCTCTAGGCTGAAGGAG
LC775 rAdcyap1r1_F	CCAGGGACTCACCTTCAGCCATAG

Supplementary Table S5. list of primer sequences.
